# Boron-Induced Electronic Modulation and Nanocrystal Fragmentation Synergistically Boost Photocatalytic Water Oxidation in Ionic Carbon Nitrides

**DOI:** 10.1021/acscatal.5c06311

**Published:** 2025-10-17

**Authors:** Haijian Tong, Valentin Diez-Cabanes, Yuanxing Fang, Guillaume Maurin, Markus Antonietti, Christian Mark Pelicano

**Affiliations:** † Department of Colloid Chemistry, 28321 Max Planck Institute of Colloids and Interfaces, Potsdam 14476, Germany; ‡ ICGM, Université de Montpellier, CNRS, ENSCM, Montpellier 34293, France; § State Key Laboratory of Chemistry for NBC Hazards Protection, College of Chemistry, State Key Laboratory of Photocatalysis on Energy and Environment, College of Chemistry, 12423 Fuzhou University, Fuzhou 350116, P. R. China; ∥ Institut Universitaire de France (IUF), Paris 75005, France

**Keywords:** ionic carbon nitrides, boron doping, nanostructure engineering, photocatalytic water oxidation, oxygen evolution

## Abstract

Photocatalytic water oxidation is a critical half-reaction to realize overall water splitting, yet it remains challenging due to sluggish reaction kinetics and the need for efficient active centers. To overcome these limitations, we introduce a dual strategy combining boron doping with nanocrystal fragmentation to boost the photocatalytic oxygen evolution reaction (OER) performance of ionic carbon nitrides. The optimal catalyst (*0.25%B*-KPHI) achieves an apparent quantum efficiency of 4.6% at 420 nm with CoO_*x*_ as a cocatalyst for OER, outperforming most previously reported carbon nitride photocatalysts. Extensive experimental analyses revealed that boron incorporation induces the fragmentation of nanocrystalline domains within the potassium poly­(heptazine imide) (KPHI) matrix, resulting in extended visible-light absorption, improved hydrophilicity, more efficient charge separation, and accelerated water oxidation kinetics. Comprehensive density functional theory calculations further showed that boron preferentially localizes at the edges of heptazine units near structural defects, where it serves as a potential adsorption site for water and substantially lowers the energy barrier for the formation of the *O intermediate.

## Graphical abstract

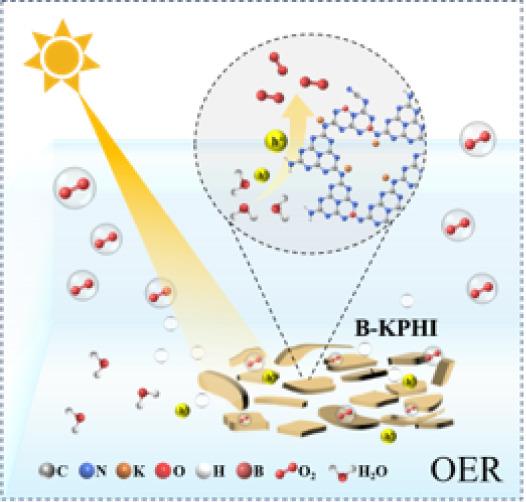

## 1 Introduction

Artificial photosynthesis offers a sustainable approach to harnessing solar energy for the sustainable production of value-added fuels, presenting a viable solution to address current global fossil crisis and environmental issues.^1,2^ Although significant progress has been made in solar-to-fuel technologies, such as solar-to-hydrogen and solar-to-organic compound conversion, their practical implementation challenges continue to limit their economic viability.^3–5^ The primary limitation lies in the sluggish photocatalytic oxygen evolution reaction (OER), which requires multiple proton-coupled electron transfers and high activation energy barrier for triplet oxygen formation, constraining the overall solar energy conversion efficiency.^6,7^ Extensive research has been dedicated to developing water oxidation photocatalysts spanning various inorganic and organic systems. However, only a limited number of photocatalysts exhibits significant efficiency for solar-driven water oxidation.

Metal poly­(heptazine imides) (*M*PHI), a class of ionic carbon nitrides, have gained significant attention as highly efficient photocatalysts for solar fuel production.^8–12^ By precisely tuning key photophysical and chemical properties, PHI has achieved outstanding apparent quantum yields for H_2_ evolution and H_2_O_2_ production, particularly in the UV range.^13,14^ Additionally, PHI possesses an exceptionally high valence band (VB) potential of ∼+2.6 eV owing to its well-structured local packing.^15^ This elevated potential provides a sufficient overpotential to drive water oxidation, enabling O_2_ evolution even in the absence of noble metal cocatalysts. This discovery challenged the existing paradigm, as previous semiconductor photocatalysts either failed to produce O_2_ or required noble metal cocatalysts.^16^ Building on these successes, a major challenge now lies in enhancing their solar absorption capabilities. Expanding the optical response of MPHI catalysts deeper into the visible spectrum is essential to improving solar energy conversion efficiency and advancing their practical deployment.

Various strategies have been explored to enhance the water oxidation capability of carbon nitrides, including heteroatom doping, defect engineering, heterojunction formation, and cocatalyst loading.^17–20^ Unlike single-metal catalysts, nonmetal doping offers an environmentally friendly and low-cost strategy to incorporate nonmetal heteroatoms in photocatalysts. This process tailors the electronic band structure of carbon nitrides by introducing midgap states, enhancing light absorption and charge separation.^21^ Wang et al. pioneered B doping in the “melon” structure, revealing its catalytic potential for the oxidation of activated aliphatic C–H bonds.^22^ Their study showed that when B atoms substitute C, strong Lewis acidic sites are created, which work synergistically with the intrinsic basic nitrogen sites to enable bifunctional catalysis.^22,23^ In another study, B atoms were directly integrated into the heptazine units of porous C_3_N_4_ via B–N coordination, leading to the formation of additional active sites. This structural modification was established to significantly increase the thermodynamic driving force for OER.^24,25^ At the same time, defect engineering has proven to be a versatile and effective method for augmenting photocatalytic conversion efficiency. Introducing surface defects, such as surface vacancies (carbon or nitrogen vacancies) and surface functional groups (cyano or amino), greatly improves both the selectivity and activity of photocatalytic reactions.^26^ These earlier findings indicate that incorporating dopants or defects into carbon nitrides enhance the activity. However, in such cases, the modifications centered only on narrowing the band gap but VB level remained unchanged or was shifted upward, even restricting the driving force for water oxidation.^27–29^ Therefore, it is crucial to achieve both a smaller band gap for better visible-light absorption and a downshifted VB to effectively facilitate the water oxidation reaction. Moreover, most existing studies have predominantly examined the isolated effects of either dopants or defects,^30,31^ particularly within covalent carbon nitride systems, while the combined impact on photocatalytic performance has not been performed.

In this work, we present a strategic approach to enhance the photocatalytic OER performance of ionic carbon nitrides by simultaneously introducing B dopants and refining the nanostructure. B incorporation serves a dual role: it promotes the fragmentation of KPHI into smaller nanocrystalline domains, thereby improving hydrophilicity and surface accessibility, while also introducing structural defects that enhance light absorption and facilitate charge separation and transport. Electrochemical measurements confirm that B doping accelerates water oxidation kinetics, consistent with the observed photocatalytic performance. The optimized B-doped catalyst (*0.25%B*-KPHI), in conjunction with a CoO_*x*_ cocatalyst, achieves an apparent quantum efficiency of 4.6% at 420 nm. Density functional theory (DFT) calculations further support that the synergy between B sites and defect structures lowers the energy barrier for water oxidation reaction and improves charge carrier dynamics.

## 2 Results and Discussion

### 2.1 Materials Characterization

The photocatalysts were synthesized via a straightforward one-step thermal polymerization method (Figure 1a). In a typical procedure, 2.5 g of 5-aminotetrazole was mixed with salt of 12.5 g KCl/LiCl salt mixture (mass ratio 0.55/0.45), followed by the addition of a specific amount of sodium borohydride (NaBH_4_) as the boron source. The mixture was ground at 25 Hz for 5 min, then transferred to a crucible and annealed at 600 °C for 4 h under N_2_ atmosphere. The resulting yellowish-brown powders were designated as *x%B*-KPHI, where *x* represents the wt % of NaBH_4_ relative to 5-aminotetrazole. For comparison, pristine KPHI was fabricated under identical conditions but without NaBH_4_ (see SI for detailed description). Pristine KPHI exhibits two characteristic diffraction peaks at 8.1° and 28.1°, corresponding to the (100) and (002) crystal planes, which represent the in-plane structural packing of heptazine units and the interlayer stacking of conjugated aromatic systems, respectively (Figure 1b).^32^ Upon increasing the B doping level, a progressive reduction in the intensity of these peaks is observed, indicating that the incorporation of NaBH_4_ during polymerization disrupts the structural ordering of KPHI. Furthermore, B doping induces a shift of the (002) peak toward lower angles, implying an expanded interlayer distance attributable to the larger atomic radius of boron. At higher doping levels (>0.5 wt % NaBH_4_), the diffraction peaks become substantially broadened, indicating significant disruption of the layered structure and amorphization of the catalysts. Thermogravimetric (TG) analysis suggests that NaBH_4_ participates in the polymerization process during its decomposition (Figures S1). As illustrated in Figure 1c, pristine KPHI shows a peak at 801 cm^–1^, assigned to the out-of-plane bending mode of heptazine rings, while the peaks at 910 and 985 cm^–1^ are attributed to the vibration modes of C–N–C bonds in K–NC_2_ groups. The spectral region between 1200 and 1700 cm^–1^ is dominated by the stretching vibrations of C–N and C=N bonds, while bands at 1575 and 1650 cm^–1^ are attributed to N–H stretching from protonated primary amine (H_2_NC) and secondary amine (HNC_2_) groups, reflecting the presence of terminal and linking functionalities. A broad vibrational peak in the 3000–3500 cm^–1^ range is ascribed to the −NH_*x*_ and −OH group bonds from uncondensed amino groups and surface-adsorbed water.^33^ In *x%B*-KPHI samples, the retention of 801 cm^–1^ peak suggests that controlled B doping does not significantly alter the heptazine framework. However, a gradual reduction of the 910 and 985 cm^–1^ peaks with increasing NaBH_4_ content suggests perturbation of the C–N–C linkages, likely causing partial decomposition of the heptazine units (Figure S2). This disruption likely induces fragmentation of heptazine units, contributing to an amplified −NH_*x*_ absorption peak, as supported by the enhanced broad band signal in the 3000–3500 cm^–1^ region. Increasing NaBH_4_ content to 0.75% led to the emergence of a new peak at 1350 cm^–1^ ascribed to the in-plane stretching vibration of B–N while the peaks in the 1200–1300 cm^–1^ range disappear, indicative of boron-rich PHI domain formation at excessive doping levels.^34,35^ At lower doping levels, B–N vibrational modes are masked by overlapping signals from =N or C=N groups. Furthermore, the intensity signal for −C≡N group amplifies with increasing B content suggests the successful introduction of additional −C≡N moieties. These observations imply that NaBH_4_ mediated deprotonation of – NH_*x*_ groups during polymerization, thereby facilitating the production of −C≡N functionalities.^36^ The controlled integration of −C≡N group is known to enhance photocatalytic performance by tuning the electronic structure while simultaneously acting as anchoring sites for photogenerated charge carriers, thereby boosting the overall efficiency.^37^

**Figure 1:**
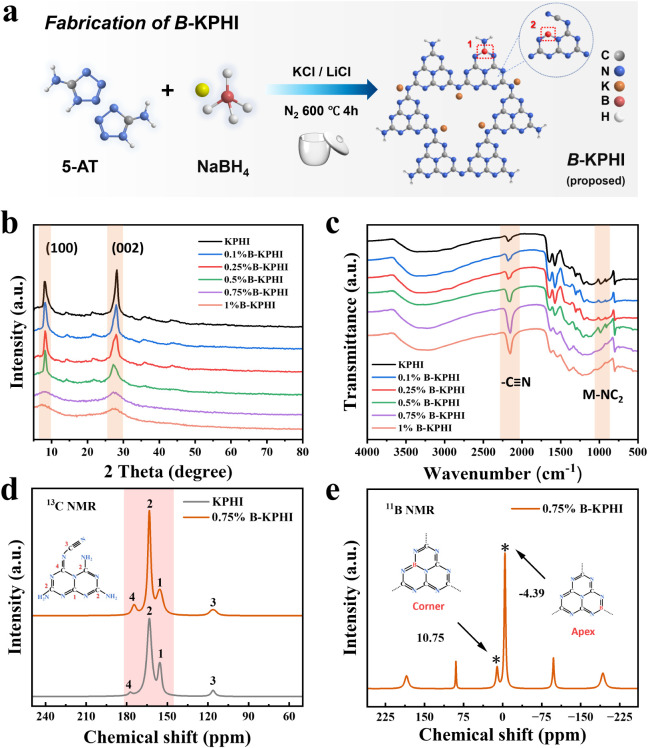
(a) Illustration for the synthesis process of *wt %B*-KPHI. (b) XRD patterns, (c) FTIR spectra, (d) Solid state ^13^C CP-MAS NMR spectra of KPHI and *0.75%B*-KPHI samples, and (e) Solid state ^11^B CP-MAS NMR spectrum of *0.75%B*-KPHI.

To further examine the chemical structure and composition of the catalysts, X-ray photoelectron spectroscopy (XPS) was performed. The full XPS survey spectra of all samples confirm the presence of C, N, O and K elements (Figure S3a). The high-resolution C 1s spectra display three distinct peaks at 284.8 eV, 286.4 and 288.1 eV, which are linked to adventitious (C–C), aminated carbons (C–NH_*x*_) at the edges of aromatic units, and sp^2^-hybridized carbon atoms (N–C=N) within the heptazine rings, respectively (Figure S3b).^38^ Notably, all the *x%B*-KPHI samples exhibit an increase in the C–NH_*x*_ peak intensity and a decrease in the N–C=N signal as the B doping level rises. Given that the binding energy of the −C≡N groups overlaps with that of C–NH_*x*_ groups,^37^ this trend aligns with the higher density of −C≡N groups observed in the FTIR analysis.

The weakening of the N–C=N signal likely stems from C atom substitution by B or, at elevated doping levels, from aggressive degradation of the heptazine framework. As previously noted, an excessive amount of NaBH_4_ significantly lowers the yield of KPHI by disrupting its π-conjugated structure, ultimately causing decomposition or amorphization of the material. The high-resolution N 1s spectra feature four binding energy peaks at 398.5 eV (bicoordinated N, C–N=C), 399.4 eV (tricoordinated N in the heptazine ring, N–C_3_), 400.7 eV (N atoms in the terminal −NH_*x*_ or – C≡N groups), and 403.7 eV (π excitation of heptazine rings) (Figure S3c).^39^ Again, the peak at 400.7 eV unveils a stronger signal in *x%B*-KPHI catalysts, indicating an increase in −C≡N groups as a result of NaBH_4_ modification. A higher NaBH_4_ concentration leads to a reduction in the N_2_C/N_3_C ratio, denoting the formation of N vacancies (Table S1). Additionally, elemental analysis reveals a systematic decrease in both C and N content with increasing B content, validating the structural modifications induced by B doping (Table S2). The boron content increased markedly from 0.38 wt % in *0.5%B*-KPHI to 1.85 wt % in *1%B*-KPHI, primarily due to the reduced incorporation of C and N caused by the higher amounts of NaBH_4_ used during synthesis.

The high-resolution B 1s XPS spectra confirm the inclusion of B into the KPHI structure (Figure S4a). Additionally, two distinct binding energies at 190.9 and 192.2 eV are observed in the *1%B*-KPHI, corresponding to B occupying apex and corner sites, respectively (Figure S4b).^23,40^ To further examine the molecular structures of the catalysts, solid-state ^13^C and ^11^B cross-polarization magic angle spinning nuclear magnetic resonance (CP-MAS NMR) measurements were conducted. In the ^13^C NMR spectrum, both KPHI and *0.75%B*-KPHI present two prominent peaks at 155.7 ppm (C_1_) and 163.3 ppm (C_2_), which are assigned to the C atoms in *N*=C–N_2_ (CN_3_) units of the heptazine ring and the sp^2^-hybridized *N*=C–N­(NH_*x*_), respectively (Figure 1d).^41^ The resonance peak at 116.2 ppm (C_3_) corresponds to the −C≡N groups,^42^ with its intensity in *0.75%B*-KPHI being higher than in pristine KPHI, consistent with the FTIR results. The peak at 174.5 ppm (C_4_) is attributed to the C adjacent to −C≡N groups, where B doping enhances its intensity.^43^ To further elucidate the influence of defects, electron paramagnetic resonance (EPR) spectroscopy was conducted on KPHI and *0.25%B*-KPHI samples (Figure S5). Both spectra display a single Lorentzian signal centered at a *g*-value of 2.003, characteristic of unpaired π-electrons delocalized within the heptazine rings. Notably, the EPR signal intensity increases significantly after B doping, indicating a higher concentration of unpaired electrons.^44^ The ^11^B NMR spectrum of *0.75%B*-KPHI displays two signals at −4.39 and 10.75 ppm, corresponding to B substitution at the apex and corner sites within the KPHI framework (Figure 1e).^42^ Taken together, these results strongly support the successful and simultaneous integration of B dopants and N defects within the KPHI framework.

The morphologies and structural features of the synthesized materials were analyzed by scanning electron (SEM) and transmission electron microscopy (TEM). As illustrated in Figure S6a, pristine KPHI exhibits well-defined nanorod structures. The introduction of NaBH_4_ during thermal polymerization influenced the crystallization process, yielding smaller crystallites (Figure S6b–d). A higher B doping concentration (*B* ≥ 0.75%) further promoted particle aggregation, leading to the formation of larger microspherical assemblies (Figures S6e and S6f). High-resolution TEM images show that the catalysts undergo a distinct morphological evolution, transitioning from nanorods (∼50 nm width) to finer rod-like structures (∼25 nm width) before ultimately developing into aggregated spherical architectures (Figure S7). An increasing B content induces a systematic decrease of crystallinity (Figure 2d–f), as confirmed by XRD results. In particular, when the NaBH_4_ content exceeds 0.5 wt %, the KPHI framework becomes predominantly amorphous, suggesting significant destruction of their crystalline structure. A closer inspection reveals that KPHI exhibits lattice fringes with a spacing of 0.31 nm, corresponding to the interlayer distance of the (002) crystal planes (Figure S8a). Upon B incorporation, this interlayer expands slightly, as evidenced by the values observed for *0.25%B*-KPHI (0.32 nm) and *1%B*-KPHI (0.33 nm) in Figures S8b and 8c. This indicates that the interlayer spacing can further increase as more B atoms, which have a larger atomic radius, are introduced into KPHI framework. Elemental mapping images show a uniform distribution of C, N and B across the nanostructures (Figure S9).

**Figure 2:**
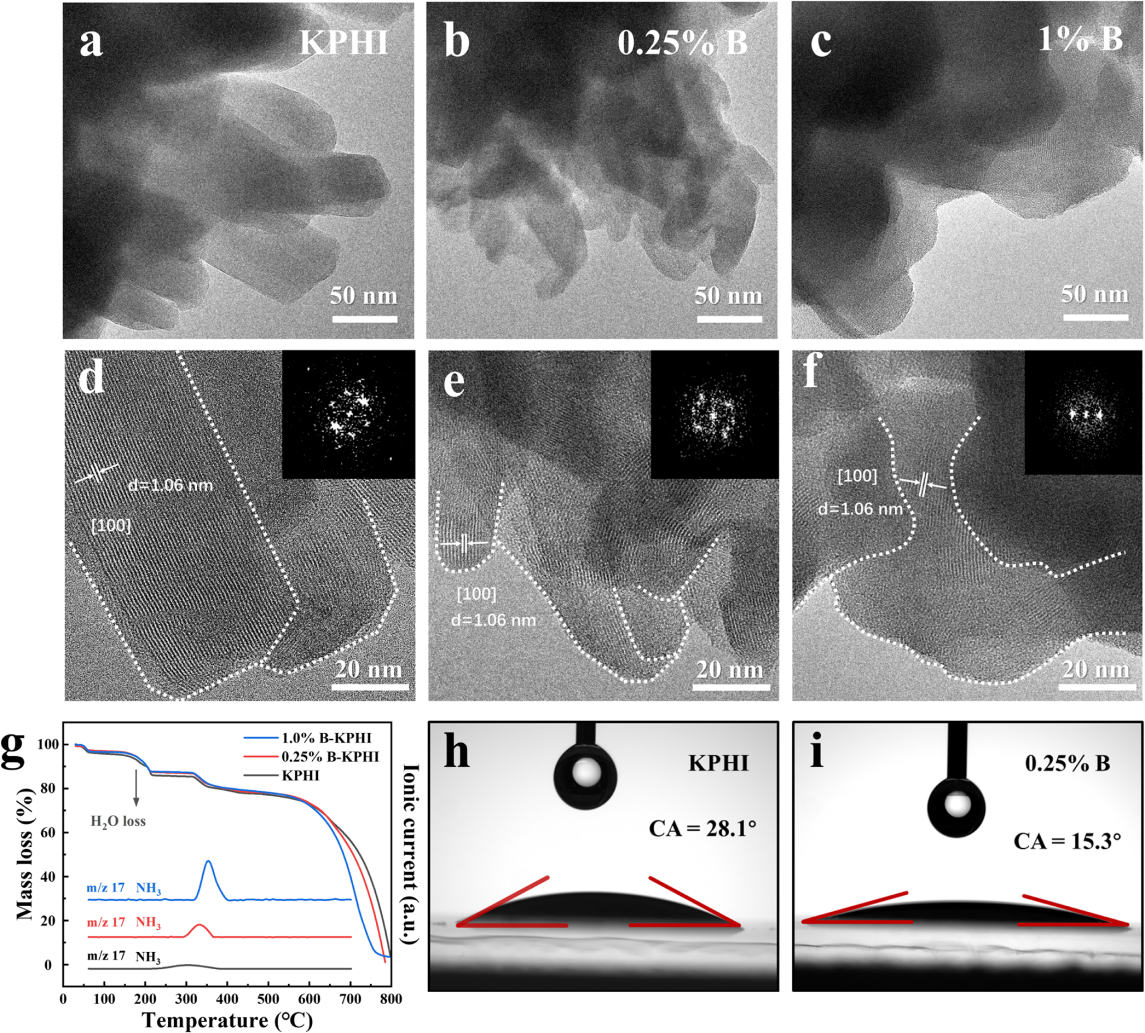
HRTEM images of (a), (d) KPHI, (b), (e) *0.25%B*-KPHI and (c), (f) *1%B*-KPHI. (g) TGA-MS of KPHI, *0.25%B*-KPHI and *1%B*-KPHI, and water contact angle images of (h) KPHI and (i) *0.25%B*-KPHI.

The observed morphological transformations were supported by the TG analysis coupled with mass spectroscopy (MS) profiles of KPHI and *0.25%B*-KPHI recorded during the thermal decomposition of their precursors (Figure 2g). The initial weight loss plateau is mainly attributed to the evaporation of residual water from synthesis stage. This is followed by another mass reduction, corresponding to the release of NH_3_ (*m*/*z* 17), which originates from the pyrolysis of terminal NH_*x*_ groups during polymerization.^45^ The introduction of NaBH_4_ significantly enhances the evolution of NH_3_ gas, with this effect being particularly pronounced in the *1%B*-KPHI sample. Previous studies have demonstrated that H_2_ can react with lattice N, creating N vacancies.^46^ As a strong reductant, NaBH_4_ undergoes thermal decomposition, generating active hydrogen species that facilitate thermal hydrogenation,^47^ thereby increasing NH_3_ release and inducing additional N defects in *x%B*-KPHI catalysts. At pyrolysis temperatures reaching 600 °C or higher, the mass loss in *x%B*-KPHI becomes substantial, lowering the final yield of the material. Low-temperature N_2_ adsorption–desorption isotherms were performed to elucidate the structural effects of B incorporation in KPHI. Excessive B doping (>0.25 wt %) leads to a marked decrease in both specific surface area and pore size, which is unfavorable for photocatalytic performance (Figure S10 and Table S3). Furthermore, water contact angle (WCA) measurements show that the WCA for *0.25%B*-KPHI (15.3°) is lower than that of pristine KPHI (28.1°), indicating improved hydrophilicity upon B doping (Figure 2h,i). This enhancement is attributed to the increased presence of surface −NH_*x*_ groups, which is in accordance with FTIR analysis. Overall, these findings confirm that the one-step thermal condensation method effectively introduces B atoms into the KPHI framework, inducing a higher density of N defects, the formation of −C≡N moieties, and extensive structural fragmentation.

### 2.2 Photocatalytic Performance

To evaluate the photocatalytic performance of the synthesized catalysts, we conducted OER measurements using AgNO_3_ as the electron sacrificial agent. AgNO_3_ has been widely employed as a benchmark in previous several relevant studies (Table S4). Hence, it was used in this work to enable direct comparison with literature-reported systems and to provide a reproducible reference point. Although AgNO_3_ is not suitable for long-term use due to Ag deposition during the reaction, it serves as an effective model electron acceptor to probe fundamental photocatalytic activity.^48^ As shown in Figure 3a, the photocatalytic activity was assessed across the full wavelength spectrum. Among all samples, *0.25%B*-KPHI loaded with 1 wt % CoO_*x*_ exhibited the highest O_2_ evolution rate, achieving a maximum rate of 34.78 μmol h^–1^ (11 μmol h^–1^ in the absence of CoO_*x*_, Figure S11) which is 1.5-fold greater than that of pristine KPHI. However, when the B doping concentration exceeded 0.5 wt %, a marked reduction in photocatalytic activity was observed. Adjusting the cocatalyst concentration to 0.5 wt % further boosted the activity to 43.49 μmol h^–1^ (Figure 3b).

**Figure 3:**
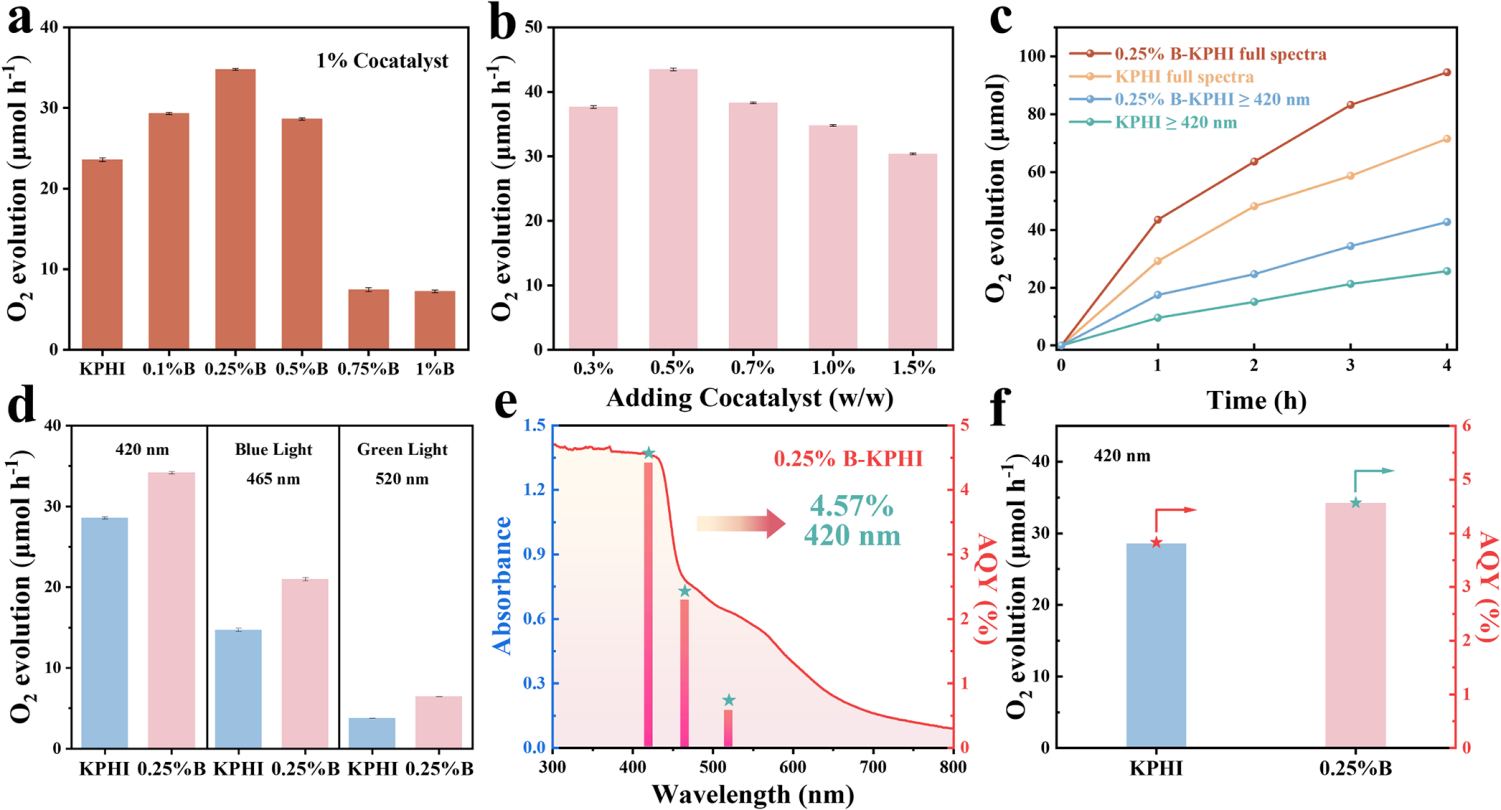
(a) Photocatalytic OER rates of KPHI and *x%B*-KPHI with 1 wt % CoO_*x*_ under full spectra UV–vis light irradiation. (b) Photocatalytic OER performance of *0.25%B*-KPHI with varying amount of cocatalysts. (c) Amount of O_2_ produced by KPHI and *0.25%B*-KPHI under full spectra and λ ≥ 420 nm. (d) Photocatalytic OER activities of KPHI and *0.25%B*-KPHI using different excitation wavelengths. (e) UV–vis spectrum and AQY values for *0.25%B*-KPHI as a function of the irradiation wavelength. (f) The oxygen evolution performance and AQY values of KPHI and *0.25%B*-KPHI with 0.5 wt % CoO_*x*_.

By comparing the reaction activity across different wavelengths, it was observed that *0.25%B*-KPHI displayed the highest performance, achieving 43.49 μmol h^–1^ under full-spectrum illumination and 7.49 μmol h^–1^ under visible light (*λ* ≥ 420 nm) after 1 h. In contrast, KPHI demonstrated lower activity, with values of 29.19 μmol h^–1^ and 9.58 μmol h^–1^ under the same conditions. After 4 h, the OER activity of *0.25%B*-KPHI further increased to 94.43 μmol h^–1^ and 42.69 μmol h^–1^, which represent 1.32-fold and 1.66-fold increases compared to KPHI (71.49 and 25.74 μmol h^–1^) under full-spectrum irradiation and visible light (λ ≥ 420 nm), respectively (Figure 3c). The observed decrease in O_2_ evolution rate upon prolonged use of AgNO_3_ is attributed to the photoreduction of Ag^+^ to Ag^0^, which deposits on the catalyst surface and acts as a recombination center and light barrier.^49^ This not only impairs photon absorption and charge separation but may also deactivate active sites, collectively leading to suppressed photocatalytic activity. The evolution curves are thereby to be judged mostly by the primary evolution rate. The optimal catalyst (*0.25%B*-KPHI) unveiled respectable durability and stability. A slight decrease in activity was observed after two consecutive cycles (8 h), which can be associated with the accumulation of reduced sacrificial agent byproducts on the catalyst surface, leading to the passivation of active sites (Figure S12).

To elucidate the relationship between light absorption and photocatalytic efficiency, wavelength-dependent O_2_ evolution experiments were conducted for *0.25%B*-KPHI and pristine KPHI (Figure 3d). Under irradiation with a 420 and 465 nm LED sources, the *0.25%B*-KPHI catalyst exhibited a 20% and 43% increase in O_2_ evolution rate compared to its undoped counterpart, respectively. Even at a longer excitation wavelength of 520 nm, the optimal catalyst maintained appreciable activity (6.46 μmol h^–1^), demonstrating a remarkable 71% improved performance with respect to KPHI (3.78 μmol h^–1^). The apparent quantum efficiency (AQY) values for *0.25%B*-KPHI reached 4.57% at 420 nm and 0.74% at 520 nm (Figure 3f), surpassing those of KPHI (Figure S13 and Table S5) and most of the reported carbon nitride-based catalysts (Table S4). Most significantly, the optimal catalyst exhibits outstanding performance even under illumination wavelengths exceeding 450 nm, a feat rarely attained by conventional carbon nitrides.

### 2.3 Optical and Electrochemical Characterization

To unravel the underlying mechanism responsible for the enhanced photocatalytic OER activity following B doping, we carried out a series of optical and photo­(electro)­chemical characterizations. *0.25%B*-KPHI catalyst shows a considerable enhancement in visible-light absorption, accompanied by a pronounced redshift (Figure 4a). This spectral shift is ascribed to n → π* electronic transition, facilitated by the presence of electron lone pair at defect sites.^50^ This activation originates from a synergistic effect involving an increased density of N defects, the formation of −C≡N groups, and optimal nanostructural refinement induced by NaBH_4_ during synthesis. However, as discussed earlier, excessive B doping causes significant structural reorganization and particle agglomeration, which detrimentally impact the optical properties. This phenomenon manifests as a decline in light absorption capacity, complemented by a noticeable blue shift in the absorption profile. The observed trend is visually reflected in the material’s coloration, as shown in the inset, where the catalyst initially deepens in color before progressively fading with higher B doping levels. As shown in Figure 4b, photoluminescence (PL) measurements at an excitation wavelength of 380 nm reveal a distinct emission peak at 500 nm for pristine KPHI, which arises from radiative carrier recombination. Upon B incorporation, the emission intensity is quenched significantly, implying that B doping generates structural defects that act as trapping sites, thereby suppressing recombination and enhancing charge separation. However, when the B content exceeds 0.25%, a noticeable increase in PL intensity is observed. This is associated with extreme structural disorder that impedes charge transport, leading to elevated radiative recombination rates. Solid-state time-resolved fluorescence measurements reveal that NaBH_4_ treatment reduces the average carrier lifetime (τ_ave_) from 1.35 ns in pristine KPHI to 1.03 ns in *0.25%B*-KPHI (Figure 4c), signifying faster charge carrier capture at active sites.^51^

**Figure 4:**
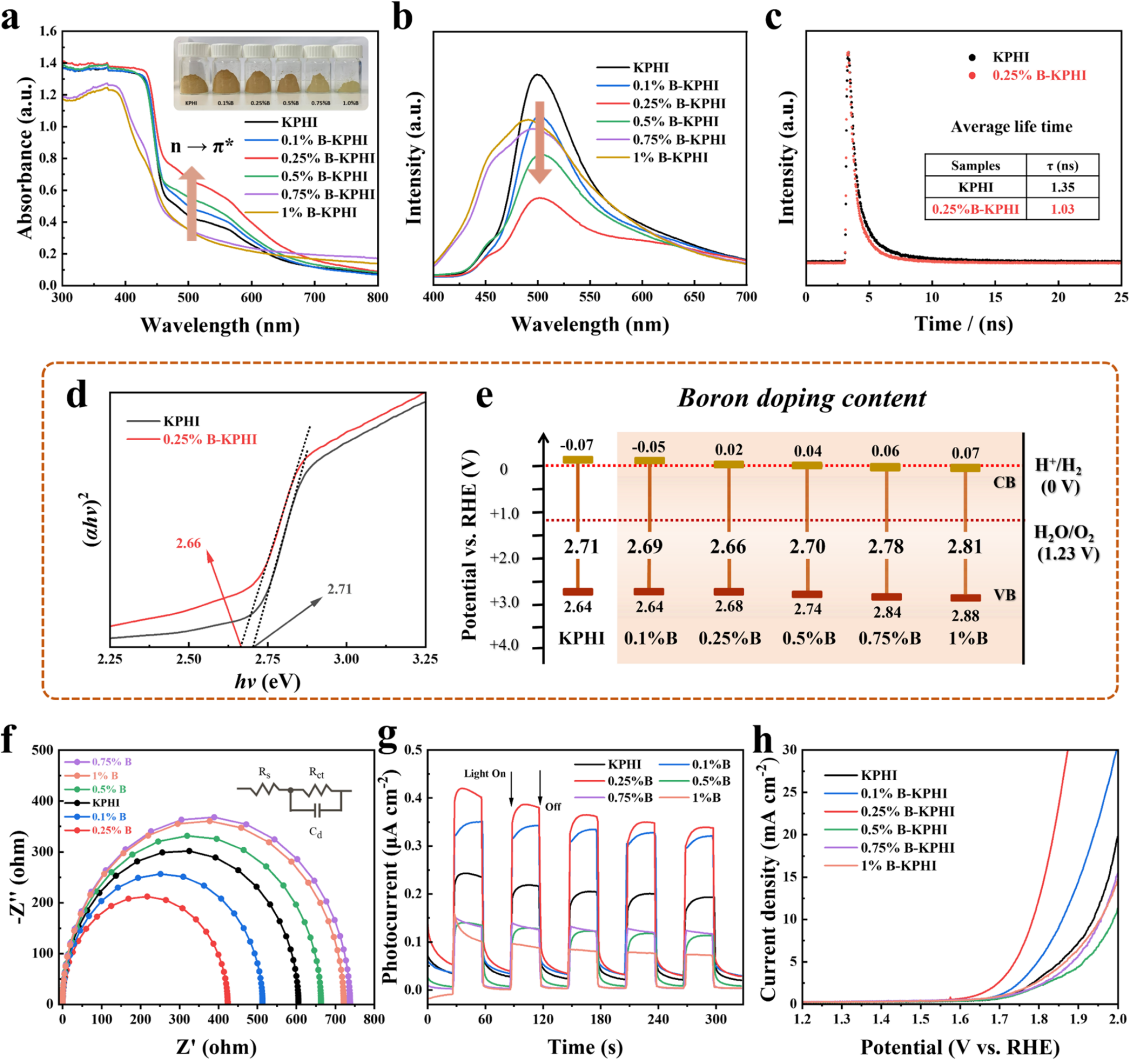
(a) UV–vis DRS spectra of KPHI and *x%B*-KPHI catalysts (inset: optical images). (b) Room temperature steady-state PL emission spectra of KPHI and *x%B*-KPHI. (c) Solid-state time-resolved PL decay of KPHI and *0.25%B*-KPHI. (d) Tauc-plot for determining the optical band gap (*E*_g_) for KPHI and *0.25%B*-KPHI. (e) Experimentally determined band structures of KPHI and *x%B*-KPHI catalysts. (f) Electrochemical impedance spectroscopy (EIS) Nyquist plots. (g) Transient photocurrent (λ = 410 nm) for KPHI and *x%B*-KPHI catalysts in 0.5 M Na_2_SO_4_ aqueous solution. (h) LSV curves of KPHI and *x%B*-KPHI catalysts in N_2_-saturated 0.5 M Na_2_SO_4_ aqueous solution.

To further clarify the differences in photocatalytic behavior between KPHI and *0.25%B*-KPHI, TRPL lifetime measurements were conducted under different experimental conditions in aqueous suspensions, i.e., in the presence of electron scavenger (KIO_3_) under Ar-saturated atmosphere (Figure S14). Under all tested conditions, *0.25%B*-KPHI consistently exhibited shorter τ_ave_ than KPHI, suggesting more efficient utilization of photogenerated carriers for H_2_O oxidation (Table S6). With addition of KIO_3_, both catalysts showed an increase in τ_ave_ (τ_ave_ = 1.40 ns for *0.25%B*-KPHI and τ_ave_ = 1.81 ns for KPHI), ascribed to the electron-accepting role of KIO_3_. By scavenging photogenerated electrons, KIO_3_ reduces recombination rates, thereby extending carrier lifetimes and ultimately promoting the oxidation reaction.^52^ Moreover, Tauc-plot analysis shows that the introduction of B causes a slight reduction in the band gap (*E*_g_), decreasing from 2.71 eV for KPHI to 2.66 eV for *0.25%B*-KPHI (Figures 4d and S15). However, when the B content exceeds 0.5%, the *E*_g_ increases, following a similar trend observed in light absorption behavior. By combining the calculated *E*_g_ values with Mott–Schottky analysis (Figure S16), the precise band edge positions of all catalysts were determined (Figure 4e). Both the conduction and valence band edges show a positive shift in potential, which augments the oxidative power of photoexcited holes and strengthens the thermodynamic driving force for water oxidation reaction.

Electrochemical impedance spectroscopy (EIS) measurements were carried out at 0 V vs the Ag/AgCl electrode to further explain the charge carrier dynamics within the *x%B*-KPHI catalysts (Figure 4f, inset). A smaller semicircle diameter in the Nyquist plot signifies a reduced *R*_ct_, indicating improved carrier transfer efficiency.^53^ It can be observed that *0.25%B*-KPHI shows the smallest semicircle, verifying its enhanced conductivity owing to B incorporation. This outcome is further supported by transient photocurrent response measurements recorded at 0.3 V vs the reference electrode under 410 nm LED illumination (Figure 4g). Among all samples, *0.25%B*-KPHI shows the highest photocurrent response of ∼0.35 μA cm^–2^, nearly 75% higher than that of pure KPHI (∼0.2 μA cm^–2^), suggesting considerably improved charge transfer. Furthermore, the surface reaction kinetics occurring over KPHI and *0.25%B*-KPHI systems were evaluated by linear sweep voltammetry (LSV). The electrocatalytic O_2_ evolution are measured in N_2_-saturated 0.5 M Na_2_SO_4_ aqueous solution (Figure 4h). A significantly higher anodic current was observed for *0.25%B*-KPHI compared to KPHI under identical potential range, indicating that B incorporation and defect engineering accelerate the O_2_ evolution kinetics on the photocatalyst surface. These photo­(electro)­chemical results align well with the observed photocatalytic performance, reinforcing the beneficial impact of NaBH_4_ modification on charge transfer and surface reaction dynamics.

### 2.4 Theoretical Exploration

The critical role of B sites on the photocatalytic OER activity of *B*-KPHI catalysts was examined using DFT calculations (see ESI sections S7.1 and S7.2 for computational details). Initially, the pristine KPHI structure and different *B*-KPHI structure models, varying in both dopant number (1 or 2 B atoms), and doping sites location (the apex and corner sites corresponding to positions 1 (B_1_) and 2 (B_2_) respectively) and separating distance between B and K^+^ (*d*_B–K_ > 7.0 Å and *d*_B–K_ < 3.8 Å associated with associated with far (f-) and close (c-) configurations, respectively, were constructed and geometry optimized. We examined especially how the relative positions of K^+^ cations and B atoms influence the stability of the *B*-KPHI structure. The optimized periodic KPHI structure consists of six heptazine units interconnected by deprotonated imide bridges (Figure 5a). Interestingly, *B*-KPHI structures where K^+^ are located near B atoms (B_c_) were consistently found to be more stable than those with K^+^ positioned farther away (B_f_), regardless of the models considered, either periodic structures (Figure S17a–d) or finite clusters (Figure S18a–d). For example, the B_2c_-KPHI model reported in Figure 5b exhibits the lowest relative energy, being 2.5 meV/atom lower than B_1c_-KPHI, identifying it as the most thermodynamically favorable configuration. Furthermore, B-doping in the corner site (B_2c_-KPHI) results in a more stable structure compared to doping at the apex site (B_2f_-KPHI, 9.8 meV/atom higher in energy). We further considered −C≡N defects in *B*-KPHI cluster models (denoted with a prime, e.g., B_2′_). The B_2′c_-KPHI configuration emerges as the most stable one (Figure 5c), exhibiting greater stability than the B_2′f_-KPHI counterpart, being 3.7 meV/atom higher in energy (Figure S18c). This further confirms that corner B-doping sites, where K^+^ ions remain in close proximity to the B atoms, are thermodynamically more favorable. Among the models doped with two B atoms, the B_12c_-KPHI was found to the most stable configuration (Figure S17f). When defects are introduced, the type I configuration of B_12′_-KPHI (Figure 5d) exhibits the highest stability compared to other variants where the two B atoms are spatially separated (Figure S18e). The electronic features of the DFT-optimized pristine KPHI and most stable *B*-KPHI structures were investigated by computing their projected density of states (PDOSs) (Figures 5e and S19). Interestingly, the introduction of B atoms primarily affects the VB edge, where their electronic states become localized, whereas the CB remains largely unchanged, showing only minor delocalization near the K^+^ cations. As a result, such a B-doping driven modification led to an average bandgap reduction of 1.02 eV for B_12c_-KPHI systems, relative to the KPHI, aligning qualitatively with the trends observed in optical bandgap measurements from Tauc-plots (Figure 4d). It is important to highlight that the quantitative discrepancy between the B doping driven bandgap reduction found at theoretical and experimental levels, arises from the fact that Tauc plot gaps are calculated by considering the band edge absorption tail, thus neglecting the darker charge transfer transitions, whereas the electronic structure gaps are estimated as the lowest energy transitions, independently if they are dark or not.

**Figure 5:**
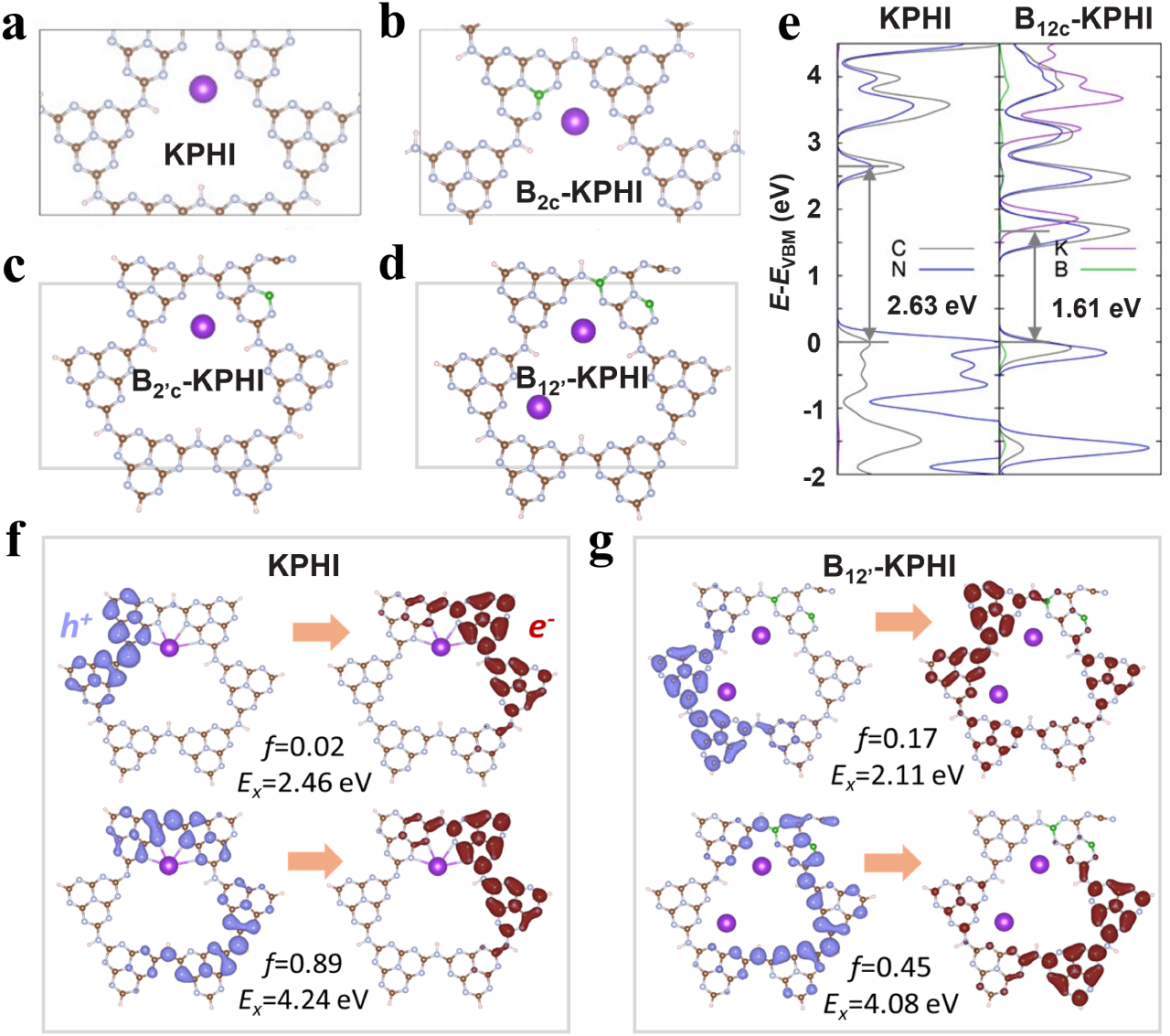
DFT-optimized (a) periodic pristine KPHI, and the most stable (b) periodic nondefective B_2c_-KPHI, (c) defective cluster B_2′c_-KPHI and (d) defective cluster B_12′_-KPHI (this last one containing B atoms located in 1 (apex) and 2 (corner) B-doping sites respectively,(e) corresponding projected density of states (PDOS) for their constituting atoms, where the valence band maximum (VBM) energy is set as energy reference. The PDOS for the rest of periodic B-KPHI systems are collected in Figure S19. Natural transition orbitals (NTO) plots for the lowest energy excited states of (f) periodic KPHI and (g) defective B_12′_-KPHI cluster spectrum indicated by the orange arrows, together with their corresponding absorption energies and oscillator strengths, respectively. The rest of NTOs for the DFT-optimized *B*-KPHI cluster models depicted in Figure S18 are represented in Figures S23–S25. Purple/red colors are used to depict the occupied/virtual NTO isodensities. The isovalue density used in the plots was set to 0.02 au, calculated at the TDA-B3LYP/DGDZVP level.

We further studied the absorption properties of the *B*-KPHI periodic systems by conducting Time-Dependent Density Functional Perturbation Theory (TD-DFPT) calculations (see ESI Sections S7.1 and S7.2 for computational details). Figure S20 shows the simulated absorption spectra for KPHI and *B*-KPHI containing one B atom. Single B-doping generally results in a slight increase in the intensity of the main π → π* KPHI absorption band located at 3.6 eV in high energy area and to the appearance of new absorption CT bands in the VIS-NIR region (low absorption energy region), in line with the trend observed in the experimental absorption spectra (Figure 4a). Furthermore, we plotted the distribution of the crystalline orbitals involved in the main transitions conforming the most relevant states from these absorption bands (Table S8). For the lowest energy absorption bands (*E*_*x*_ < 3.3 eV), hole particles are localized around the B-doping atom, whereas electrons are distributed in the aromatic cores surrounding K^+^, thus leading to a ligand-to-ligand charge transfer (LLCT) within the *B*-KPHI layer (Figure S21). In the case of the higher absorption π → π* bands both hole and carriers are fully delocalized along the *B*-KPHI layer. Finally, with the aim of evaluating the influence of B-doping in the electron–hole interaction strength, we computed the exciton binding energies (*E*_b_) (Table S9) as the difference between the electronic (*E*_g_) and the optical (*E*_S1_) gaps corresponding to the energy of the first excited state. All considered *B*-doped KPHI models containing a single boron atom exhibit almost identical exciton binding energies compared to pristine KPHI, indicating that B doping has a negligible effect on the electron–hole interactions. The excited state properties of the defective B_2′_-KPHI and B_12′_-KPHI cluster models simulated by TD-DFT calculations show an obvious adsorption peak at low energy region (Figure S22) evidencing that the presence of terminal −C≡N groups introduces new absorption features in this energy region for the *B*-KPHI systems. Therefore, in comparison to pristine KPHI, the B-doped systems featuring −C≡N defects exhibit an increased carrier generation under visible light irradiation. Furthermore, the cluster models containing two B doping atoms, such as B_12′_-KPHI, presents a wider absorption peak compared to KPHI, proving that *B*-doping and −C≡N defect formation could increase the light-harvesting ability of KPHI in VIS-NIR range, supported by the UV–vis DRS spectra results.

Natural transition orbital (NTO) analysis was conducted to examine the spatial distribution of photogenerated charge carriers and the nature of the electronic excitations (see Figures S23–S25). As pointed in our previous work, the low-energy excitations (∼2 eV) of PHI cation loaded materials are dominated by LLCT or n → π* type of transitions.^54,55^ Within this energy region, *B*-KPHI model excited states present more spatially separated electron–hole pairs, especially around defect-rich heptazine rings, implying prolonged carrier lifetime. As a general trend, the formation of −C≡N defects in *B*-KPHI models does not translate into a significant modification of the excited state nature. Only in the case of B_12′_-KPHI, holes are more spatially delocalized around the B sites while electrons are distributed across the opposite side (Figure 5g), facilitating efficient charge separation facilitates and transfer. Conversely, pristine KPHI (Figure 5f) showed both carriers confined in similar or neighboring heptazine rings, resulting in poor charge separation and stronger PL intensity (Figure 4b), detrimental to photocatalytic performance. In line with the results from the periodic materials, at higher excitation energies (∼4 eV), the transitions correspond to π → π* excitations, where both hole and electron carriers are delocalized along the aromatic framework.

To further identify the most favorable active center for OER, either the doped B atom or K^+^, the adsorption energies (*E*_ads_) of a single water molecule on both sites were calculated at the DFT level for the pristine KPHI structure and different *B*-KPHI structure models (Figure 6a–d and Table S11). Typically, the corresponding *E*_ads_ values for the active sites K^+^ and B in B_2′_-KPHI are −0.80 and −2.15 eV, associated with equilibrium separating distances from the water molecule’s oxygen atom of 3 Å and 1.5 Å respectively (Table S11). Indeed the B_2′c_-KPHI as well as B_12′_-KPHI models show significantly higher affinity at B sites, indicating that water molecules preferentially adsorb on the doped B atoms.

**Figure 6:**
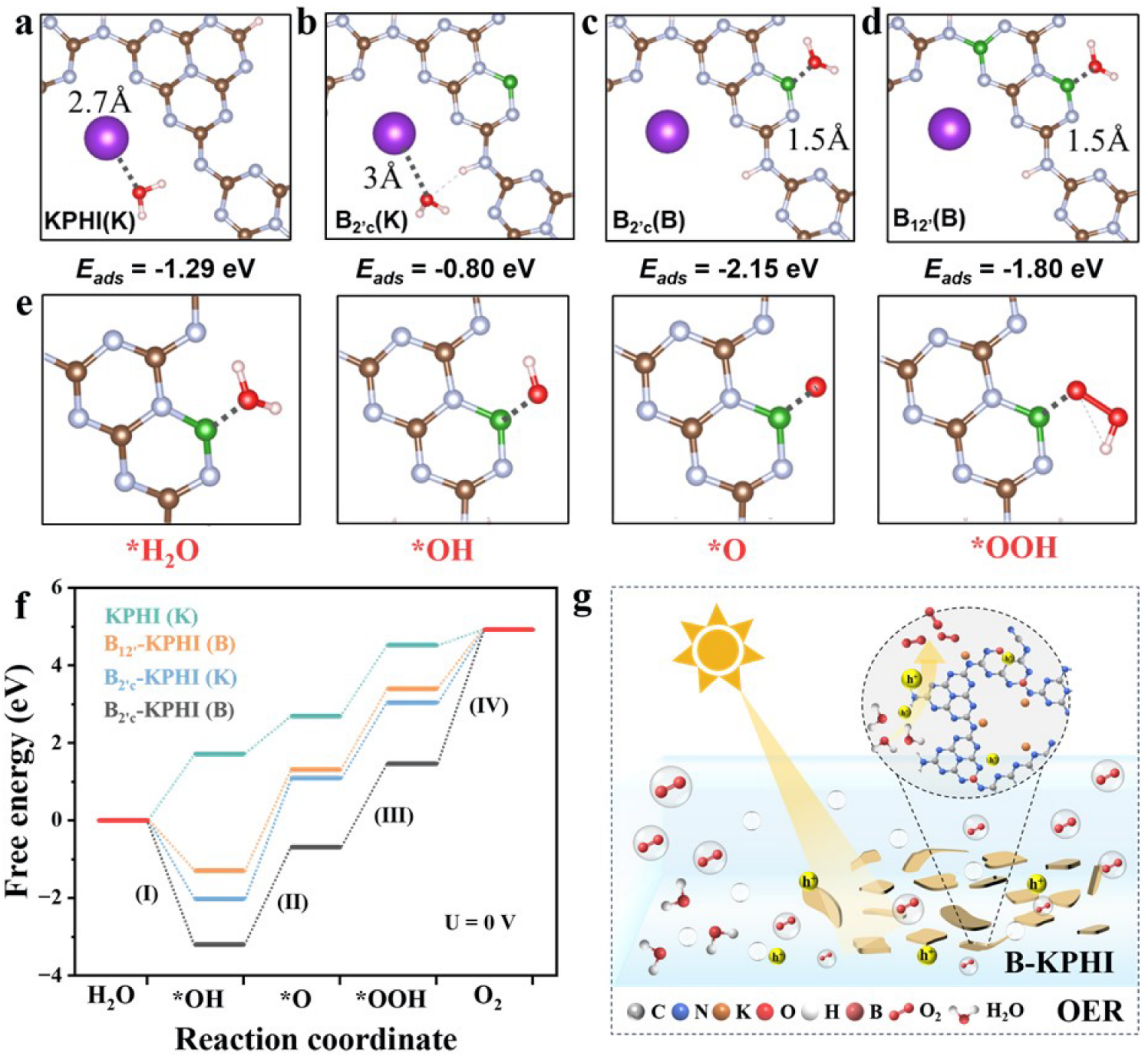
DFT-Computed OER thermodynamic properties for KPHI and *B*-KPHI cluster models: top views of a water molecule adsorbed at (a) K^+^ active sites on KPHI cluster, (b) K^+^ and (c) B active sites in B_2′c_-KPHI cluster, (d) B active sites in B_12′_-KPHI cluster, with the corresponding *E*_ads_ calculated as *E*_ads_ = *E*_PKPHI‑H2O_ – *E*_KPHI_ – *E*_H2O_, (e) detailed view of the OER intermediates adsorbed in the B site of the B_12′_-KPHI cluster, (f) Gibbs free energy profile for the OER process of KPHI, the defective B_2′c_-KPHI and B_12′_-KPHI cluster models, (g) a brief schematic of the photocatalytic mechanism of O_2_ production over *B*-KPHI.

To further elucidate the role of B-doping and defective sites in the OER mechanism of the *B*-KPHI systems, further free energy calculations were conducted. The reaction pathway proceeds through the typical sequence: (I) *H_2_O to *OH, (II) *O, (III) *OOH, and (IV) O_2_ formation and release, completing the catalytic cycle. Figure 6e typically shows the adsorption configurations of these intermediates at B sites in the B_12′_-KPHI cluster. The corresponding Gibbs free energy profiles for KPHI and the different *B*-KPHI models, considering both K^+^ and B as active sites, are presented in Figure 6f and summarized in Table S12. For B-doped active sites, whether with one or two B atoms in defective *B*-KPHI clusters, the formation of *OH intermediate (the first OER step) is highly exothermic (Δ*G* = −3.2 eV for B_2′c_-KPHI and Δ*G* = −1.3 eV for B_12′_-KPHI), in stark contrast to pure KPHI (Δ*G* = +1.72 eV), where it is endothermic and rate-limiting. Interestingly, the limiting overpotentials for both B and K^+^ sites in B_2′_-KPHI are comparable (3 eV), suggesting that the two active sites can equally contribute to the OER activity of the boron-doped KPHI systems.

The enhanced O_2_ evolution in *B*-KPHI can thus be attributed to the higher density of active sites and the greater water affinity at B sites. Moreover, the Gibbs free energy profile for B_12′_-KPHI indicates that dual B doping lowers the energy barrier for *O formation by 0.5 eV compared to K^+^ sites in B_2′_-KPHI, shifting the rate-determining step and confirming enhanced catalytic efficiency. A schematic in Figure 6g illustrates how B doping introduces nanocrystalline domains and structural defects in KPHI, promoting n → π* electronic transitions, extending light absorption, and increasing reactive site density. These structural and electronic enhancements facilitate efficient generation, separation, and transfer of photogenerated charge carriers to B active sites, thereby boosting solar-to-O_2_ conversion efficiency.

## 3 Conclusions

In summary, we designed a strategy to boost the photocatalytic oxygen evolution efficiency of ionic carbon nitrides through the concurrent incorporation of boron dopants and nanoscale structural modulation. The incorporation of NaBH_4_ during polymerization not only induces boron doping but also enables the fragmentation of the KPHI framework into finer nanocrystalline domains, thereby enhancing surface wettability and accessibility. At the same time, it generates structural defects that improve light-harvesting capabilities and promote efficient charge separation and transport, as further confirmed by TD-DFT calculations. Electrochemical characterization reveals that boron incorporation significantly speeds up water oxidation kinetics, aligning well with the enhanced photocatalytic activity. An optimal catalyst, *0.25%B*-KPHI, coupled with a CoO_*x*_ cocatalyst, delivers an AQY of 4.6% under 420 nm irradiation. Complementary DFT calculations reveal that B sites act as favorable adsorption sites for H_2_O molecules and facilitate the formation of the *O intermediate by reducing the associated energy barrier, thereby enhancing the overall solar-to-O_2_ conversion efficiency. This study provides a detailed structure–function analysis of boron-modified KPHI, elucidating how compositional and morphological tuning can significantly enhance photocatalytic oxygen evolution in ionic carbon nitride frameworks.

## 4 Methods

### 4.1 Catalyst Preparation

#### 4.1.1 Synthesis of KPHI

Potassium poly­(heptazine imide) (KPHI) was synthesized following the methodology outlined in our prior publication. ^**[1]**^ The procedure involved taking 2.5 g of dried 5-amino-1*H*-tetrazole monohydrate and 12.5 g of a KCl/LiCl eutectic mixture (in a 0.55/0.45 ratio) and placing them in a steel ball mill vessel. This mixture was then ground at an operational frequency of 25 Hz for 5 min. The resulting white powder was transferred to a porcelain crucible with a lid and heated in a furnace. The temperature of the furnace was gradually increased to 600 °C at a rate of 2.3 °C per minute under a continuous flow of N_2_ gas (4 L/min) and held at this temperature for 4 h. After the heating process, the furnace was allowed to cool down naturally to room temperature. The product was then transferred from the crucible into a beaker with deionized H_2_O and stirred at room temperature overnight. Following this, the mixture was vacuum filtered, washed extensively with water through centrifugation, and dried in a vacuum oven at 60 °C overnight.

#### 4.1.2 Synthesis of *x*%B-KPHI

The synthesis of *x%B*-KPHI followed a similar procedure to KPHI, with a minor modification in the salt template. Specifically, 0.1–1 wt % (relative to the weight of 5-amino-1*H*-tetrazole) of NaBH_4_ was added to the KCl/LiCl eutectic salt mixture to prepare B-KPHI with varying boron content. The weight ratio of NaBH_4_ to 5-amino-1*H*-tetrazole was maintained between 0.1 and 1, and the subsequent steps were identical to those for KPHI. The resulting catalysts were designated as *x%B*-KPHI, where *x* represents 0.1, 0.25, 0.5, 0.75, and 1, respectively.

### 4.2 Evaluation of Photocatalytic Performance

Photocatalytic O_2_ evolution was carried out in a Pyrex top-irradiation reaction vessel connected to a closed glass gas-circulation system. In each experiment, 50 mg of catalyst powder was dispersed in 110 mL of aqueous solution containing 0.01 M AgNO_3_ (electron acceptor) and 0.2 g La_2_O_3_ (pH buffer). Prior to initiating the reaction, *X* wt % CoO_*x*_ cocatalyst was introduced into the suspension, where *X* = 0.3, 0.5, 0.7, 1.0, or 1.5, calculated relative to the catalyst mass. Co­(NO_3_)_2_·6H_2_O was used as the precursor for CoO_*x*_ deposition. The reactant solution was evacuated several times to completely remove air prior to irradiation under a 300 W Xe lamp and a water-cooling filter. The wavelength of the incident light was controlled by using an appropriate long pass cutoff filter. The temperature of the reactant solution was maintained at room temperature by a flow of cooling water during the reaction. The evolved gases were analyzed by gas chromatography equipped with a thermal conductive detector.

## Supplementary Material



## Notes

Detailed data of the materials characterizations, XPS spectra, elemental analysis, EPR spectra, SEM and TEM images, BET measurements, photocatalytic water oxidation experiments, TRPL plots, Mott–Schottky plots, simulation models, PDOS for different systems, detailed reaction energies, absorption spectra, NTO plots (PDF)
