# Supplementary material for: Boron-Induced Electronic Modulation and Nanocrystal Fragmentation Synergistically Boost Photocatalytic Water Oxidation in Ionic Carbon Nitrides
Source: ACS Catal. 2025 Oct 17;15(21):18024–36. doi: 10.1021/acscatal.5c06311 (PMC12603997; doi:10.1021/acscatal.5c06311)
Supplement: Supplementary file 1 [file cs5c06311_si_001.pdf]

Supporting Information

Boron-Induced Electronic Modulation and Nanocrystal Fragmentation Synergistically Boost Photocatalytic Water Oxidation in Ionic Carbon Nitrides

Haijian Tong, [1] Valentin Diez-Cabanes, [2] Yuanxing Fang, [3] Guillaume Maurin, [2, 4, *]

Markus Antonietti, [1] and Christian Mark Pelicano [1, *]

[1]Department of Colloid Chemistry, Max Planck Institute of Colloids and Interfaces, Potsdam 14476, Germany

[2]ICGM, Université de Montpellier, CNRS, ENSCM, Montpellier, 34293, France

[3] State Key Laboratory of Chemistry for NBC Hazards Protection, College of Chemistry, State Key Laboratory of Photocatalysis on Energy and Environment, College of Chemistry, Fuzhou University, Fuzhou 350116, P. R. China

[4]Institut Universitaire de France (IUF), France

Corresponding author email: christianmark.pelicano@mpikg.mpg.de
Corresponding author email: guillaume.maurin1@umontpellier.fr

Experimental Section

1. Chemicals

All chemicals and solvents used were purchased from different chemical suppliers (Thermo Scientific, Merck, Sigma-Aldrich) in high purity grade and were used as received. 5-Amino-1H-tetrazole monohydrate was dried in a vacuum oven before using.

2. Characterizations

X-ray powder diffraction (XRD) patterns were measured using a Rigaku Smart Lab (Japan, Cu K, 0.154 nm) at a generator voltage of 40 kV and a generator current of 50 mA, with a scanning speed of 2 °/min from 5 ° to 80 °. X-ray photoelectron spectroscopy (XPS) spectra were performed via Thermo Fisher Scientific ESCALAB 250Xi. Fourier transform infrared (FTIR) spectroscopy measurements were conducted using a Thermo Scientific Nicolet iD5 spectrometer with an attenuated total reflection sampling technique. Thermogravimetric Analysis (TGA) was performed using Shimadzu TGA-60H thermo-balance. This apparatus

operated within a temperature range of approximately 25 to 900 °C, with a synthetic air flow rate of 50 mL/min and a heating rate of 10 °C/min in an alumina crucible. TGA-MS measurements were performed using a thermo microbalance TG 209 F1 Libra (Netzsch, Selb, Germany) coupled with a Thermostar MS (Pfeiffer Vacuum; Asslar/Germany) with an ionization energy of 75 eV. Samples were heated at 2.5 $K \cdot min^{-1}$ from 30 to 800 °C in a helium flow of 10 $mL \cdot min^{-1}$. Data were recorded and analyzed by the Proteus (6.0.0 to 8.0.0) and Quadstar (7.03, MID modus) software package. The ^{13}C and ^{11}B CP MAS spectra were recorded on a Bruker AVANCE NEO 400 WB spectrometer (Bruker BioSpin AG, Fällanden, Switzerland) equipped with a 4 mm standard bore CPMAS probehead whose X channel was tuned to 100.62 MHz for ^{13}C and the other channel was tuned to 400.18 MHz for broad band ^{1}H decoupling, using a magnetic field of 9.39T at 297 K. The dried and finely powdered samples were packed in the ZrO_2 rotor closed with Kel-F cap which were spun at 8 kHz rate. The experiments were conducted at a contact time of 2 ms. A total of 1000 scans were recorded with 6 s recycle delay for each sample. Inductively coupled plasma mass spectrometry (ICP-MS) was conducted using a PerkinElmer ICP-OES Optima 8000. Elemental combustion analysis (EA) was carried out with a vario MICRO cube CHNOS elemental analyzer from Elementar Analysensysteme GmbH. Nitrogen adsorption-desorption isotherms and pore size distributions were measured at 77 K with a Quantachrome Quadrasorb SI instrument. Prior to each measurement, the samples were degassed at 150 °C under a vacuum of 0.5 Torr for 15 hours. The specific surface area of each material was calculated from the adsorption branch data ($P/P0 < 0.3$) using the Brunauer-Emmett-Teller (BET) method. The sample morphologies were examined using a scanning electron microscope (SEM) (Zeiss LEO 1550-Gemini) with an energy-dispersive X-ray (EDX) detector (Oxford Instruments X-MAX). Transmission electron microscopy (TEM) images were captured using a JEOL JEM F200 and a double Cs corrected JEOL JEM-ARM 200F operating at 80 kV, equipped with a cold-field emission gun and a high-angle silicon drift EDX detector (Jeol JED 2300, with a solid angle up to 0.98 steradians and a detection area of 100 mm^2). The optical properties and charge carrier behaviors were analyzed using UV-vis diffuse reflectance spectroscopy (UV-vis DRS, UV-2600, Shimadzu, Japan), electron paramagnetic resonance (EPR, Bruker EMXnano), steady-state photoluminescence (PL) with a Jasco FP-8300 fluorescence spectrometer at an excitation wavelength of 365 nm and time-resolved PL (TRPL), recorded on fluorescence lifetime spectrometer (FluoTime 250, PicoQuant) equipped with PDL 800-D picosecond pulsed diode laser drive. The average lifetime (τ_{ave}) is calculated as follows:

$$\tau_{ave} = (A_1\, \tau_1^2 + A_2\, \tau_2^2 + A_3\, \tau_3^2) / (A_1\, \tau_1 + A_2\, \tau_2 + A_3\, \tau_3) \qquad \textbf{(1)}$$

3. Photoelectrochemical measurements

All photoelectrochemical measurements were carried out in a three-electrode configuration, with a Pt wire and Ag/AgCl as counter and reference electrodes, respectively. The electrocatalysis experiments were conducted with a Gamry Interface 1010E potentiostat. To prepare the working electrode, F-doped Tin Oxide (FTO) glass (3 x 1 cm) substrates were cleaned sequentially with detergent, distilled H_2O and ethanol for 15 min each to remove organic impurities. Half of the FTO area was protected with a Scotch tape. A catalyst ink was obtained by mixing 5 mg of photocatalyst powder, 0.5 mL of H_2O and 20 μL of 5 wt.% Nafion by sonication for 30 min. Then, 50 μL of catalyst slurry was pipetted onto the FTO electrode and dried at 60 °C and further heated at 120 °C for 1 h to improve adhesion. All measured potentials were converted to reversible hydrogen electrode (RHE) according to the following equation (2):

$$E_{RHE} = E_{Ag/AgCl} + 0.059 * \mathrm{pH} + 0.197 \qquad \textbf{(2)}$$

3.1 Transient photocurrent response (TPR)

The photocurrent response was measured at 0 V versus reference electrode in 0.5 M aqueous Na_2SO_4 solution under white LED (100 mW cm^{-2}) illumination using a Gamry Interface 1010E potentiostat.

3.2 Electrochemical impedance spectroscopy (EIS)

For *EIS*, the same electrodes were used as described above and the measurements were done in a frequency range of 10 kHz to 1 Hz. The data were fitted to a full semicircle using Z-View software.

3.3 Mott–Schottky measurements (MS)

MS measurements were performed in a Biologic MPG-2 system at different frequencies using the same electrodes as described above.

4. Apparent quantum yield (AQY) estimation

The AQY was measured using different monochromatic LEDs (420 nm, 465 nm and 520 nm). The AQY was obtained by the following equation (3):

$$\text{AQY (\%)} = \frac{4 * Rproduct * N_A * hc}{I * A * \lambda} * 100 \quad \textbf{(3)}$$

where $R_{product}$ is the production rate of O_2 molecules (mol s^{-1}) after the 1st hour of photocatalytic reaction, N_A is Avogadro constant (6.022×10^{23} mol^{-1}), h is the Planck constant (6.626×10^{-34} J s^{-1}) multiplied by c the speed of light (3×10^{8} m s^{-1}) giving ($1.98644586 \times 10^{-25}$ J m), A is the irradiation area (cm^2), I is the intensity of irradiation light (W cm^{-2}), and λ is the wavelength of the monochromatic light (nm).

5. Theoretical calculations

5.1 Quantum-chemical calculations at the periodic level

We first considered our previously reported periodic single K-PHI layer model (cell dimension a=21.4 Å, b=12.4 Å and vacuum of 20 Å in the c direction), containing 1 extra-framework K^+ [2] that replaces 1 proton bounded to the N atom of the pristine PHI-H layer to maintain charge neutrality. As suggested by the X-ray photoemission spectroscopy measurements, B-doping in the apex and corner sites (see **Figure 1a**), which correspond to positions 1 (B_1) and 2 (B_2) represented in **Figure S17 a-d** were considered in our study. Therefore, two periodic unit cells of B-KPHI were then constructed by replacing 1 or 2 carbon atoms by boron atoms in the single KPHI layer model. The resulting B concentrations for the 1- and 2-substituted single layer models amounted to 1.34 wt. % and 2.55 wt.%, respectively, which are slightly overestimating the highest B concentration considered experimentally (1 wt. %). Note, however, that these single layer models represent the best compromise between reproducing the experimental concentrations of both K and B doping atoms, and keeping the model as small as possible in order to employ highly accurate electronic structure methods (i.e. hybrid DFT functionals). For 1 substituted-B atom (in positions 1 or 2), we reintroduced the H-atom in the PHI-layer to keep charge neutrality, whereas for the scenario of two B-atoms (both 1 and 2 positions (B_{12})) since

no more N atoms can be protonated and in order to keep the B/K ratio constant, a second K^+ was introduced (see **Figure S17**). With the aim of investigating the role played by the separating distance between the incorporated K^+ and the B-doping atoms we have analyzed two scenarios, where both B and K^+ remain far (-f) ($d_{B\text{-}K} > 7.1$Å) or close (-c) ($d_{B\text{-}K} < 3.8$Å) from each other (see **Table S8**). Indeed, B-KPHI models containing 1 B atom in the case of K^+ is next to B (case -c) were found about 10 meV/atom lower with respect to the scenario with K^+ is far to B atom (case -f). This led to the construction of six B-KPHI systems displayed in **Figure S17**. These B-KPHI models were then geometry-relaxed at the Density Functional Theory (DFT) by using the generalized gradient approximation (GGA) Perdew−Burke−Ernzerhof (PBE) exchange-correlation functional, [3] and the Grimme's D3 dispersion method to consider the van der Waals interactions. [4] We adopted a plane-wave basis set with an energy cutoff of 550 eV, whereas a Monkhorst−Pack scheme with a 2 × 3 × 1 k-point grid was employed for sampling the First Brillouin zone. The atomic positions were fully relaxed at fixed cell parameters until they reached a convergence criterion equal to 10^{-5} eV and 0.01 eV/Å for the energies and forces, respectively. All DFT relaxations were conducted by adopting projector augmented wave (PAW) pseudopotentials as implemented in the Vienna Ab initio Simulation Package (VASP) code. [5, 6]

The opto-electronic properties of the geometry-optimized B-KPHI models were then assessed by employing the hybrid functional B3LYP with a ground state electron density energy cut-off equal to 600 Ry. Vertical excitations were computed by relying on the Time-Dependent Density Functional Perturbation Theory (TD-DFPT) linear response approach [7] within an energy cut off of 200 Ry for the excited state electronic density, and an energy convergence threshold of 10^{-5} eV. Valence and core electrons were represented by a Double-Zeta Valence Polarized (DZVP) MOLOPT basis set and Goedecker−Teter−Hutter (GTH) pseudopotentials, respectively. [8, 9] All this set of calculations were carried out at the Γ point within the CP2K package. [10]

5.2 Quantum-chemical calculations at the cluster-level

The photocatalytic behavior of the B-KPHI materials bearing edge defects (appearance of C≡N groups) was investigated by adopting the same computational strategy as the one developed in our previous works.[2] Finite cluster models were obtained by cutting one representative B-PHI-M ring from the relaxed periodic structures, and by terminating the unsaturated sites by H atoms. For the cluster models the B concentrations were equal to 0.94 wt. % and 1.81 wt.% for

the 1- and 2-B substituted systems, thus being closer to the experimental B doping concentrations when compared with the periodic single layer models. Note that, for the sake of comparison, their corresponding non-defective B-KPHI cluster models have been investigated as well. To be in line with the exploration of the periodic systems, we also considered the two scenarios were B and K^+ are far or close to each other (scenarios -f and -c respectively). As it was the case for the periodic models, the proximity of B atom and K^+ results in a stabilization of the clusters amounting to 10.8 and 3.7 meV/atom for the pristine and defective B-KPHI clusters, respectively (see **Figure S18**). Being consistent with the methodology employed to compute the electronic structure of their periodic structures, the resulting clusters were optimized at the DFT level by using the B3LYP functional, a DGauss Double-Zeta Valence Polarized (DGDZVP) basis set, [11] and the Grimme's D3 dispersion method to consider the van der Waals interactions dispersion method to treat the vdW interactions. [4] Firstly, the vertical excitations for the relaxed clusters were estimated by relying on Time-Dependent DFT (TD-DFT) calculations performed at the same level of theory as the ground state one. In line with our previous works, Tamm-Dancoff Approximation (TDA) was applied to reduce the computing time and the effect of multiplet instabilities. [12] A full-width-high-maximum width of σ=0.15 eV was set to compute the absorption spectra. The energy barriers for the OER reaction were obtained by calculating the Gibbs' free energy (G) of the reaction intermediates, which are formed via proton/electron ($H^+ + e^-$) transfers following the associative mechanism proposed by Rossmeisl et al. [13] The four-electron oxidation reaction implies following elementary steps:

OH* formation:	$2H_2O(l) + * \rightarrow OH^* + H_2O(l) + (H^+ + e^-)$	**(4)**
O* formation:	$OH^* + H_2O(l) \rightarrow O^* + H_2O(l) + (H^+ + e^-)$	**(5)**
OOH* formation:	$O^* + H_2O(l) \rightarrow OOH^* + (H^+ + e^-)$	**(6)**
O_2 formation:	$OOH^* \rightarrow O_2(g) + * + (H^+ + e^-)$	**(7)**

The adsorption energy E_{ads} of the OER intermediates were calculated as follows:

$$E_{ads} = E_{system} - (E_{catalyst} + E_M) \quad \textbf{(8)}$$

where M stands for OH*, O* or OOH* reaction intermediates and E_M their corresponding ground state energies. The computed overpotential (μ) were estimated by using computational

hydrogen electrode approach established by Nørskov et al. [14] Within this approach, the Gibbs free energy variations (ΔG) for each elemental step were calculated as follows:

$$\Delta G = \Delta E + \Delta ZPE - T\Delta S \quad \textbf{(9)}$$

where E is the reaction energy, ZPE and S are the zero point-energy and entropy corrections, as computed via frequency calculations, whereas T was set to 298.15 K. The whole set of cluster model calculations were conducted within the Gaussian16 package. [15]

6. Supplementary data

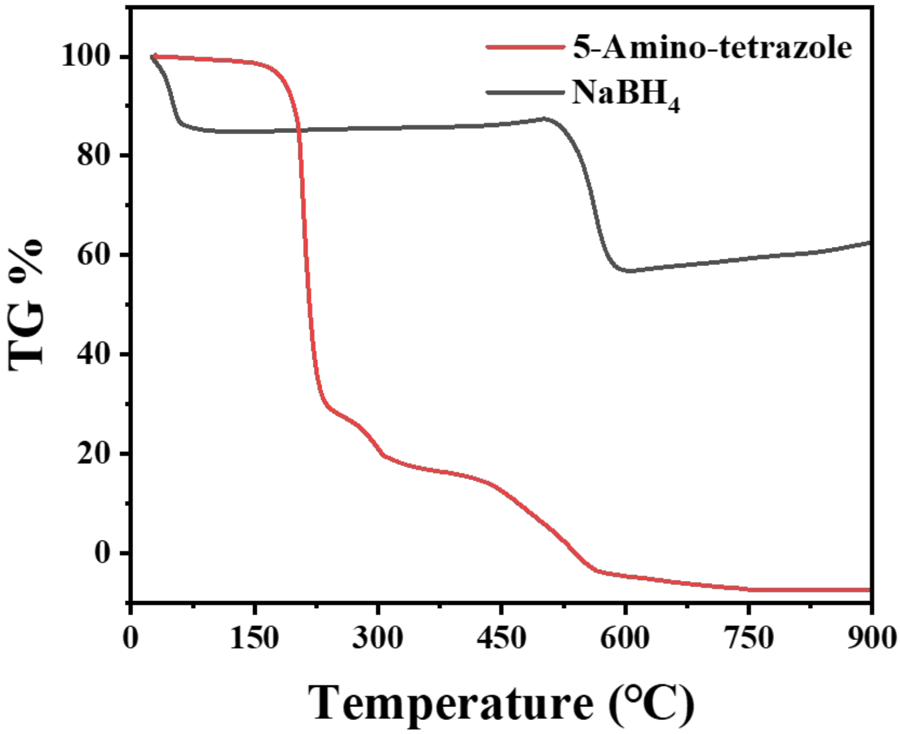

Figure S1. TG curves of 5-Amino-tetrazole and $NaBH_4$ recorded under helium atmosphere (10 K min^{-1}).

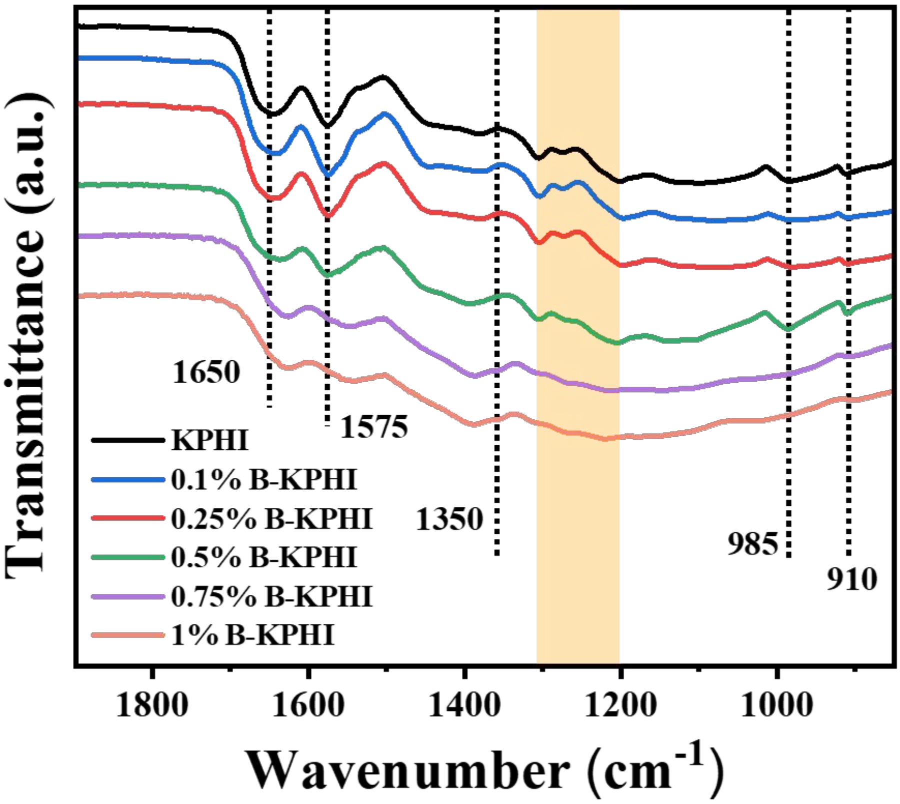

Figure S2. Magnified FTIR spectra of all samples.

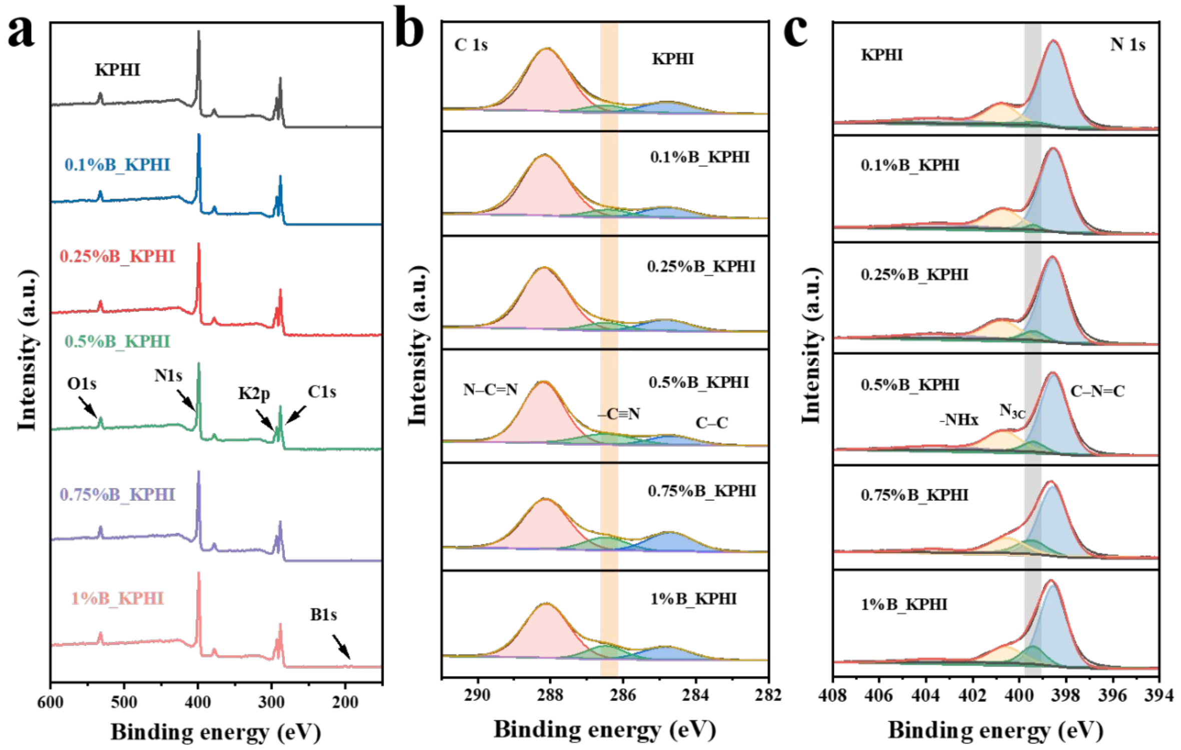

Figure S3. (a) XPS full spectra, (b) high-resolution C 1s and (c) N 1s of KPHI and *x%* B-KPHI.

Table S1. XPS N atom ratio of all samples.

Samples	N_{2C}	N_{3C}	N_{2C}/N_{3C}
KPHI	0.77	0.17	4.53
0.1% B-KPHI	0.72	0.18	4.00
0.25% B-KPHI	0.67	0.19	3.53
0.5% B-KPHI	0.65	0.20	3.25
0.75% B-KPHI	0.63	0.21	3.00
1% B-KPHI	0.62	0.23	2.69

Table S2. The relative ratios of C, N and H elementals in all samples determined by EA and the B element and the total amount of alkali metals in all catalysts via ICP.

Samples	C (%)	N (%)	H (%)	B (%)	K (%)	Li (%)
KPHI	27.20	45.75	2.20	-	11.1	0.26
0.1% B-KPHI	27.08	45.82	2.10	0.04	10.1	0.26
0.25% B-KPHI	27.30	45.40	2.13	0.08	11.2	0.27
0.5% B-KPHI	26.65	44.61	2.07	0.38	12.1	0.51
0.75% B-KPHI	25.22	44.03	2.15	0.94	11.4	0.74
0.1% B-KPHI	24.58	43.63	2.05	1.85	12.2	0.83

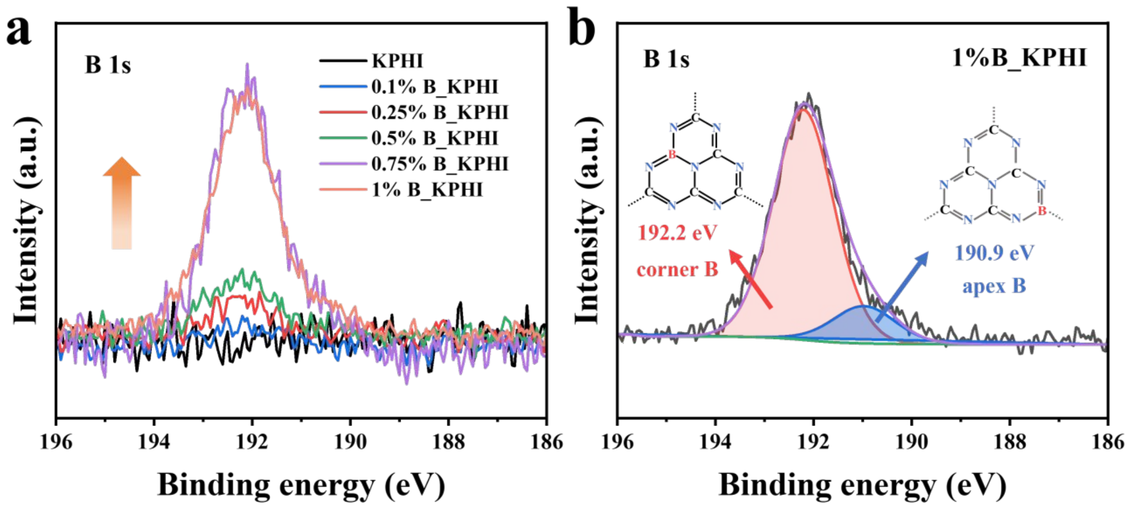

Figure S4. (a) High-resolution B 1s XPS spectra of KPHI and *x%* B-KPHI and (b) B 1s XPS fitting of *1%* B-KPHI.

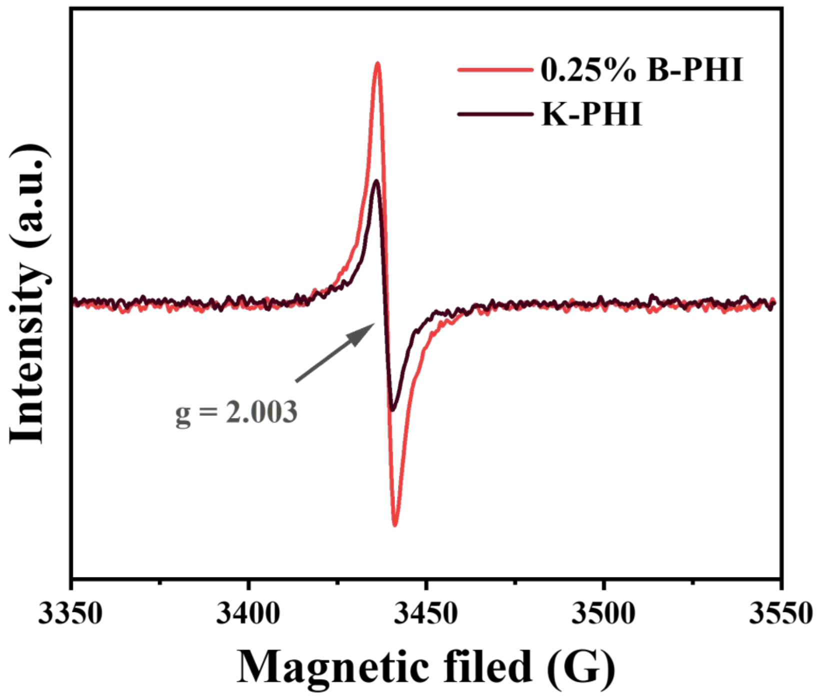

Figure S5. EPR signals of KPHI and 0.25%B-KPHI.

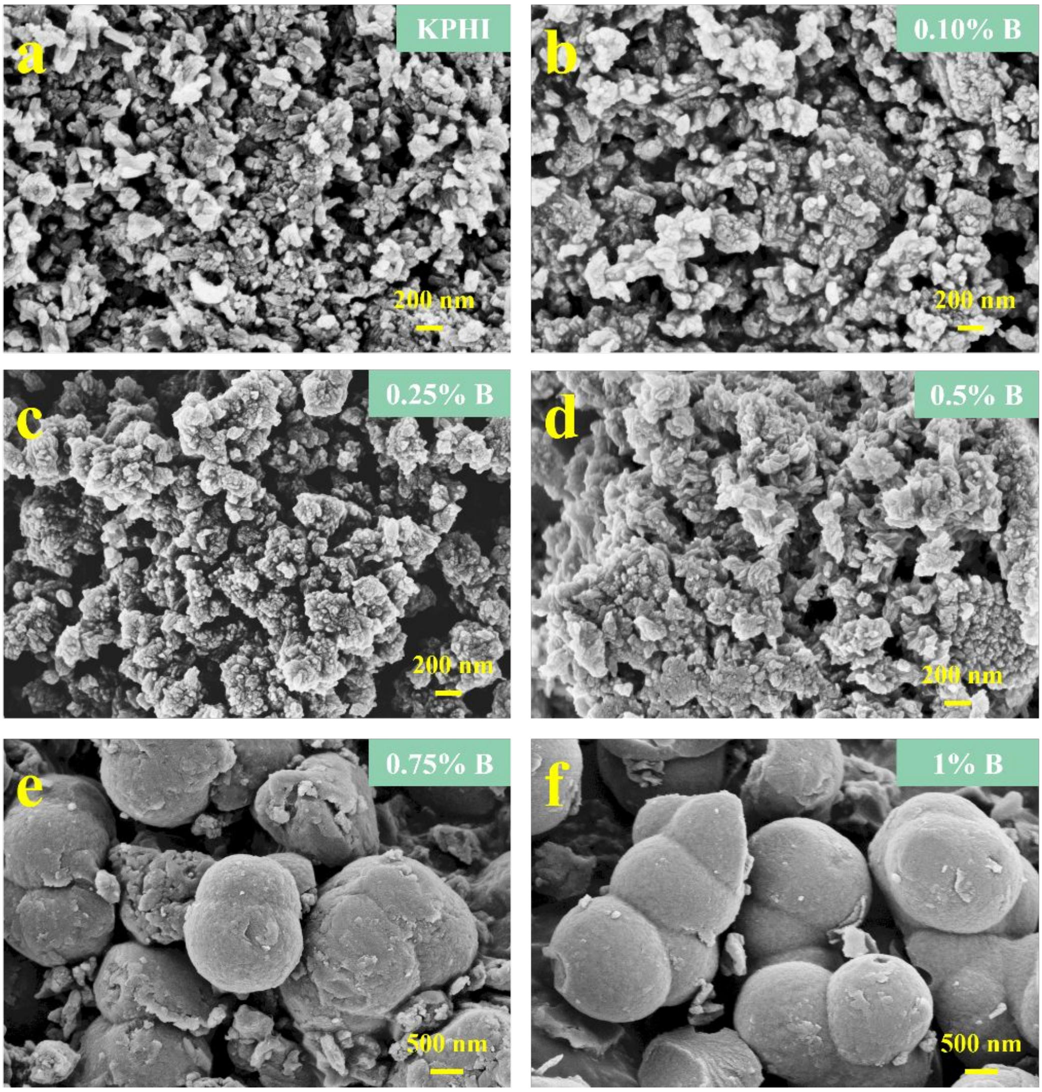

Figure S6. SEM images of pristine KPHI and all *x%* B-KPHI catalysts.

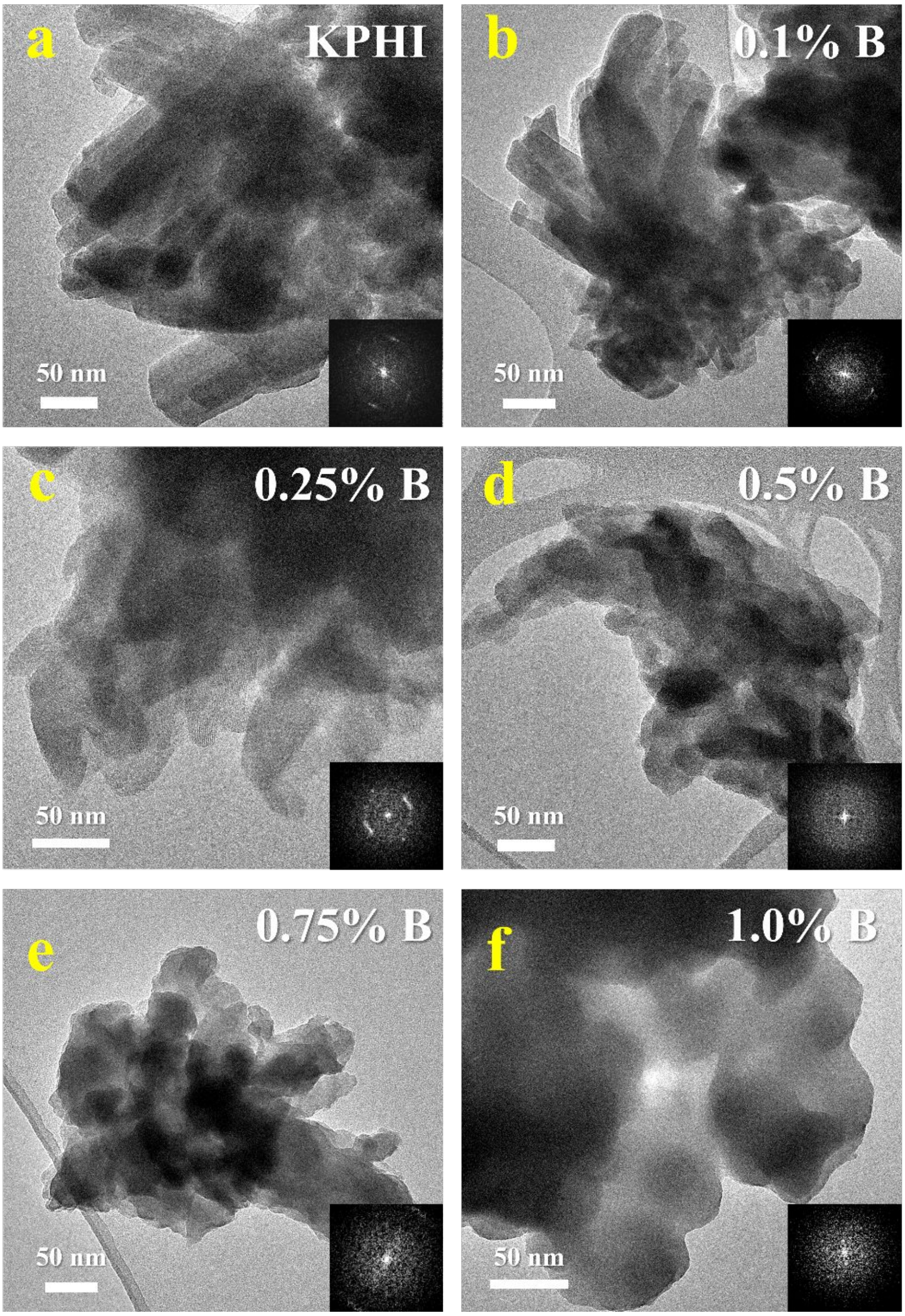

Figure S7. High-resolution TEM images of pristine KPHI and *x%* B-KPHI catalysts.

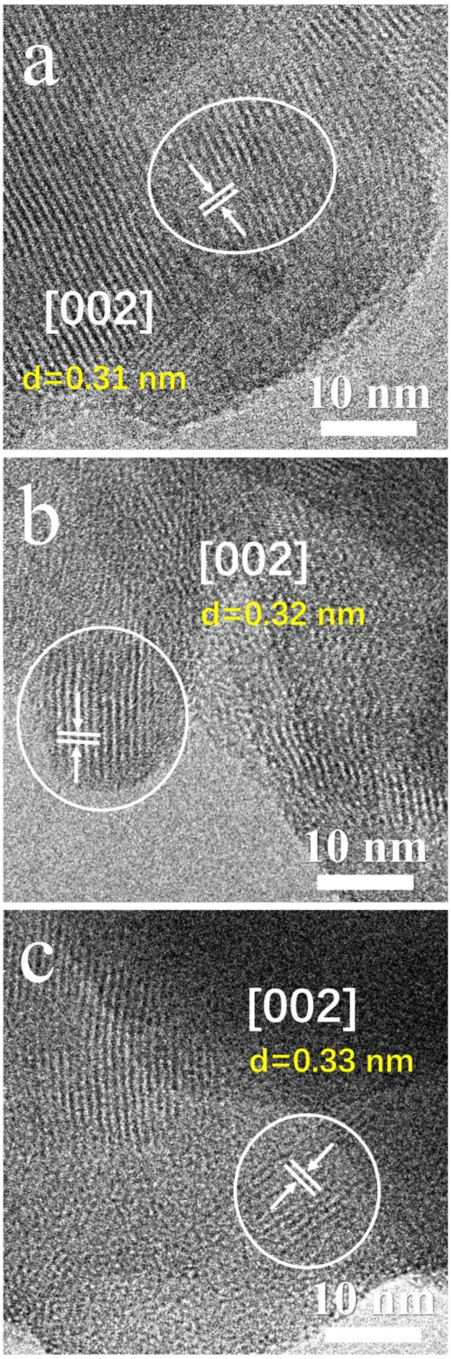

Figure S8. HRTEM images of (a) KPHI, (b) *0.25%B*-KPHI and (c) *1%B*-KPHI.

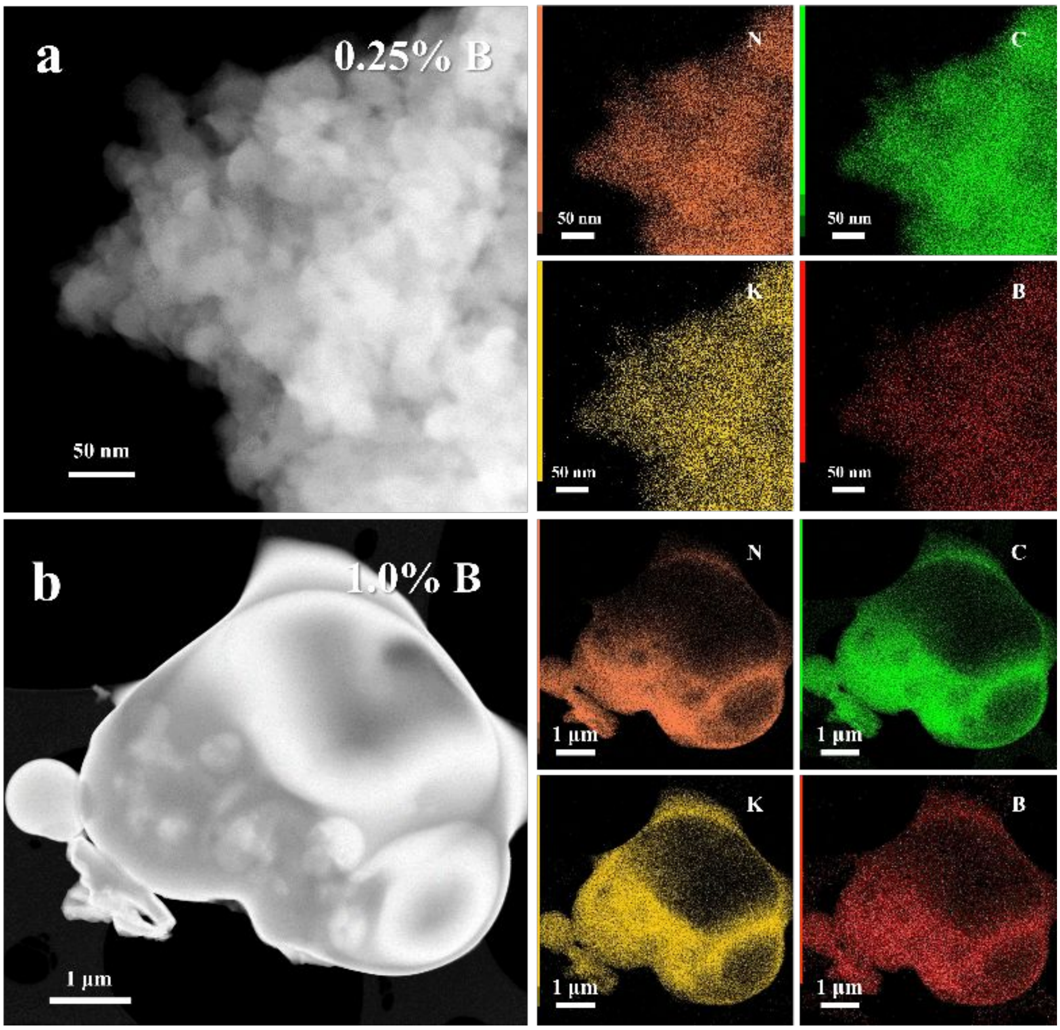

Figure S9. High-angle annular dark-field scanning TEM (HAADF-STEM) images of (a) *0.25%*B-KPHI and (b) *1%*B-KPHI and their corresponding elemental mapping images.

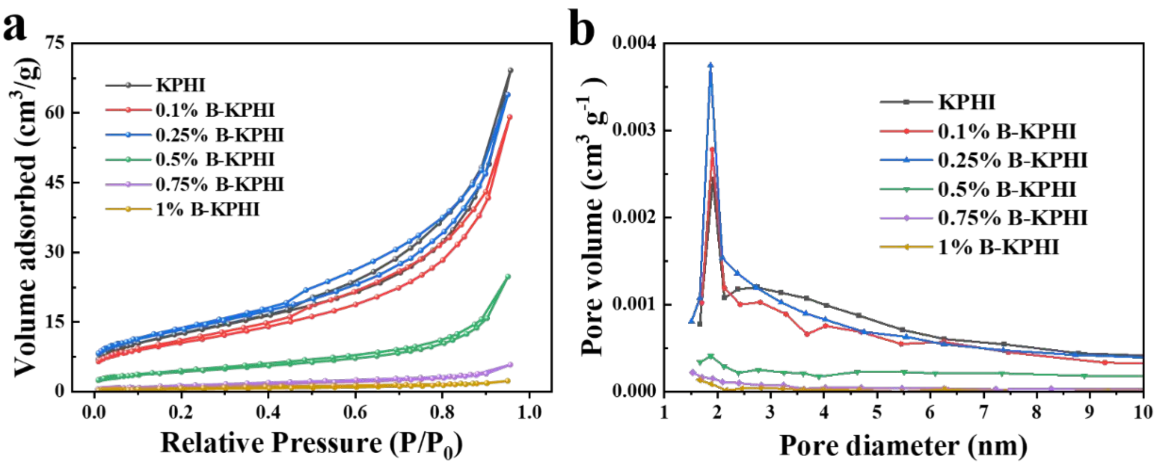

Figure S10. (a) N_2 adsorption/desorption isotherm at 77K and (b) BJH pore size distribution from N_2 adsorption branch at 77K of KPHI and *x%* B-KPHI.

Table S3. BET specific area, pore volume and diameter size of all prepared samples.

**Sample**	**S_{BET} m^2 g^{-1}**	**V_p cm^3 g^{-1}**	**D_p nm**
KPHI	45.574	0.102	1.909
0.10% B-KPHI	42.940	0.087	1.902
0.25% B-KPHI	47.697	0.093	1.866
0.50% B-KPHI	15.351	0.035	1.873
0.75% B-KPHI	4.324	0.008	1.533
1.00% B-KPHI	1.949	0.003	1.657

Table S4. Comparison of oxygen evolution production yields and AQY values with other carbon nitride photocatalysts

Photocatalyst	Modification	Synthesis	Sacrificial agents	Conditions	O_2 yields (μmol g^{-1} h^{-1})	AQY (%)	Ref.
g-C_3N_4	-	Calcination	0.01M $AgNO_3$	Xe-lamp (λ>400nm)	11.8	-	**16**
P-g-C_3N_4	Protonation	HCl-treated	0.01M $AgNO_3$	Xe-lamp (λ>400nm)	45.3	2.1% 410 nm	**16**
Co_3O_4/g-C_3N_4	Co_3O_4 hybrid	Calcination	0.01M $AgNO_3$	Xe-lamp (λ>420nm)	502	1.1% 420nm	**17**
g-C_3N_4-CM_{10}	$CoMn_2O_4$ hybrid	Immersed	0.01M $AgNO_3$	Xe-lamp (λ>300nm)	366	1.0% 380 nm	**18**
NiCoP@NiCo –Pi/g-C_3N_4	Core/shell structure	Grinding	0.02M $AgNO_3$	UV-vis light (full spectra)	312	0.6% 420nm	**19**
BH400	B-doped N-deficient	Calcination	0.01M $AgNO_3$	UV-vis light (λ>300nm)	561.2	3.7% 380 nm	**20**
g-C_3N_4 /Cs_xWO_3	CW compounds	electrostati c attraction	0.01M $AgNO_3$	UV-vis-NIR light (full spectra)	266.5	1.56% 420 nm	**21**
Co-PCN	single-atom modification	Calcination	0.1M $AgNO_3$	Xe-lamp (visible light)	746	2.06% 420 nm	**22**
PCN-B-20	Boron doping	Post-calcination	0.01M $AgNO_3$	Xe-lamp (λ>420nm)	248.9	5.8% 420 nm	**23**
KPHI	-	Calcination	0.01M $AgNO_3$	Xe-lamp (λ>420nm)	191.52	3.8% 420 nm	**this work**
0.25%B-KPHI	Boron doping	Calcination	0.01M $AgNO_3$	Xe-lamp (λ>420nm)	349.79	4.6% 420 nm	**this work**

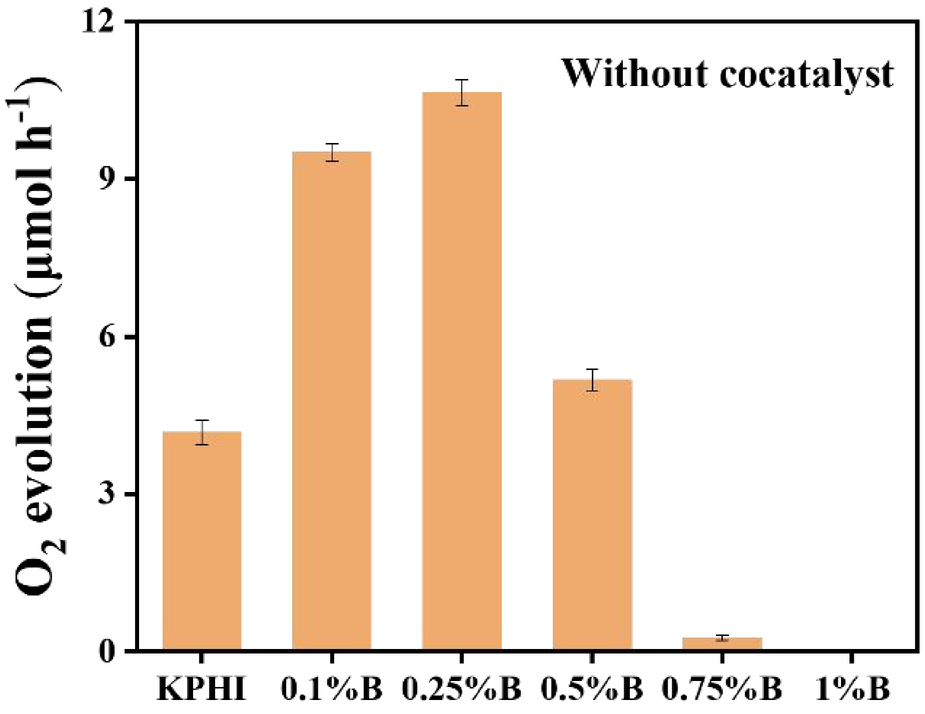

Figure S11. Photocatalytic oxygen evolution reaction performance of KPHI and all boron modified samples without cocatalyst.

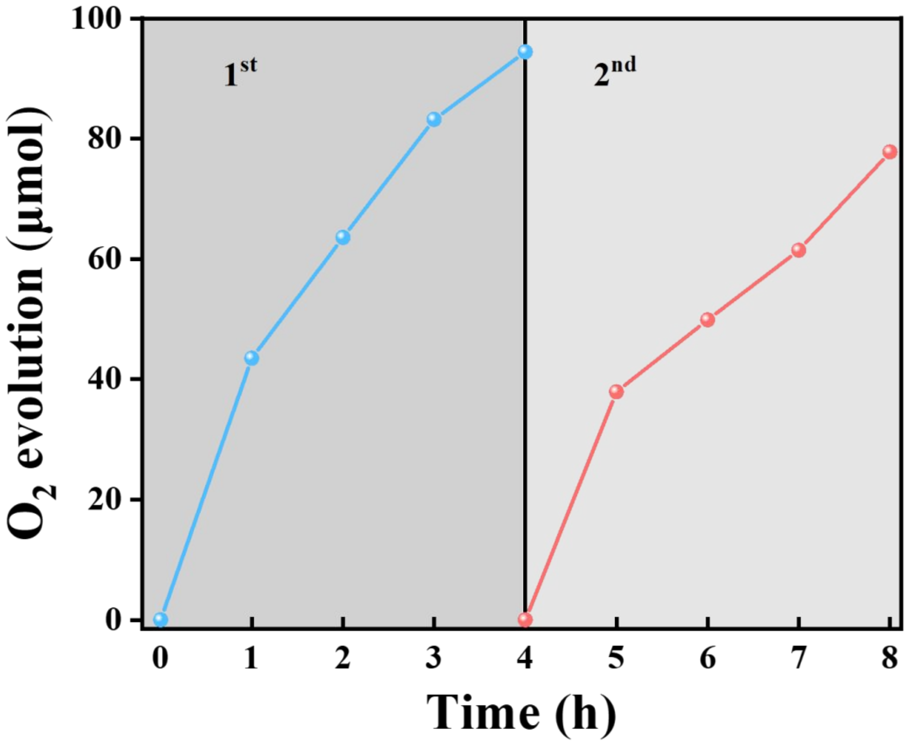

Figure S12. The recycling oxygen evolution reaction photocatalytic performance of 0.25% B-KPHI with $AgNO_3$ electrons sacrificial agent.

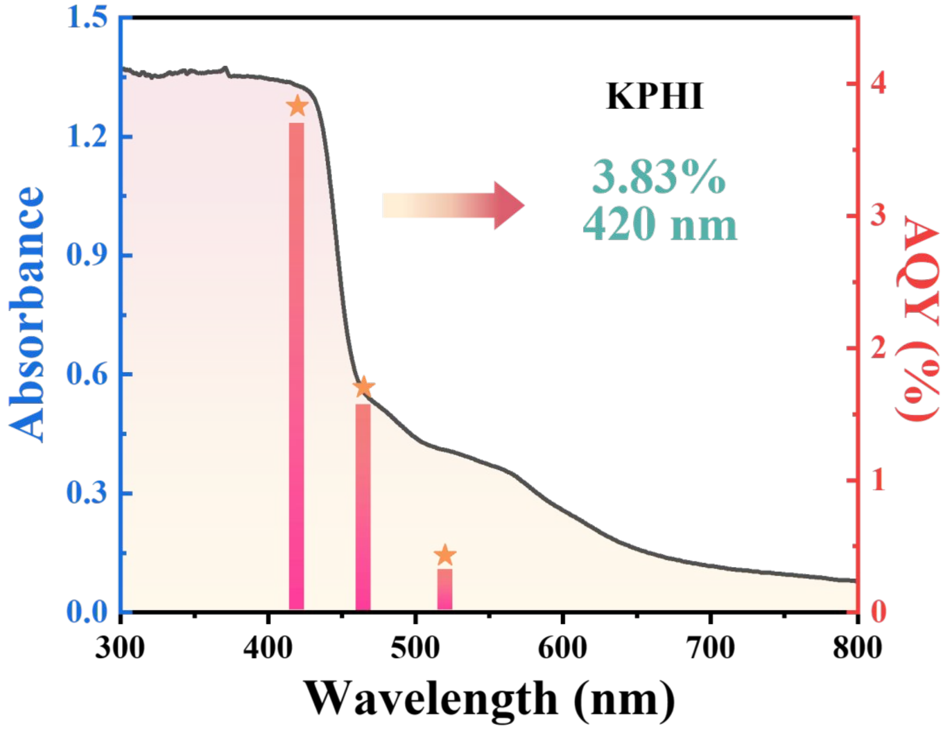

Figure S13. UV-vis spectrum and AQY values for KPHI as a function of the irradiation wavelength.

Table S5. The AQY values of KPHI and 0.25%B-KPHI under different wavelengths.

Wavelength (nm)	AQY_{KPHI}	$AQY_{0.25\%B\text{-}KPHI}$
420	3.83	4.57
465	1.97	2.81
520	0.43	0.74
420 (without cocatalyst)	0.82	1.33

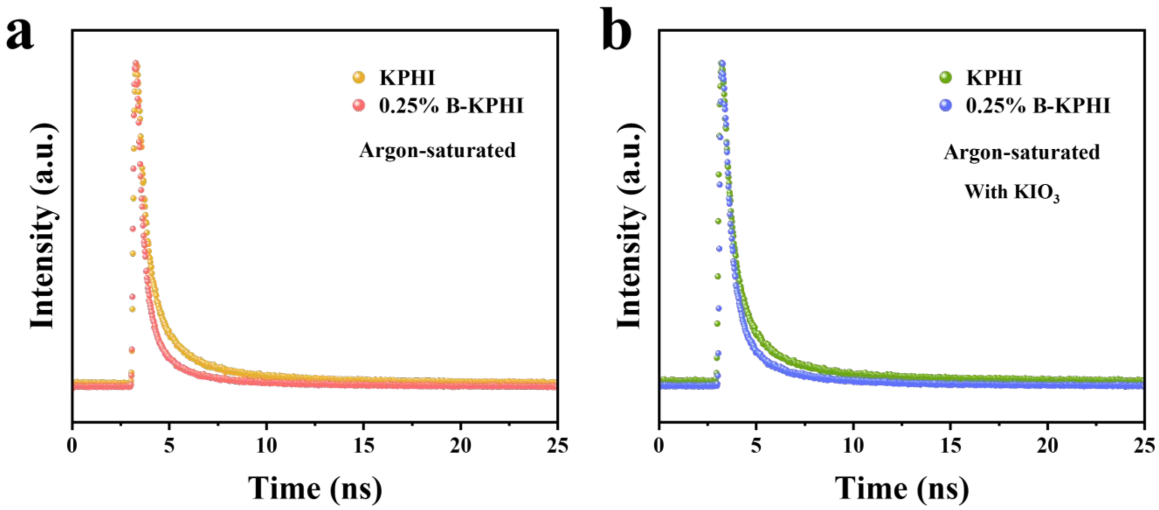

Figure S14. Time-resolved transient PL decay curves of KPHI and 0.25%B-KPHI in aqueous suspensions with or without KIO_3 purged with Argon.

Table S6. PL lifetime values of KPHI and 0.25%B-KPHI in aqueous suspension under different experimental conditions with argon purged.

Sample	Solution	A_1	τ_1 (ns)	A_2	τ_2 (ns)	A_3	τ_3 (ns)	τ_{ave} (ns)	Purged Gas
KPHI	H_2O	26.88	0.11	6.29	0.81	1.24	3.83	**1.77**	Ar
KPHI	with KIO_3	25.02	0.12	6.37	0.84	1.19	3.98	**1.81**	
0.25%B	H_2O	53	0.06	6.9	0.56	1.06	3.16	**1.24**	
0.25%B	with KIO_3	37	0.09	6.19	0.65	1.02	3.44	**1.40**	

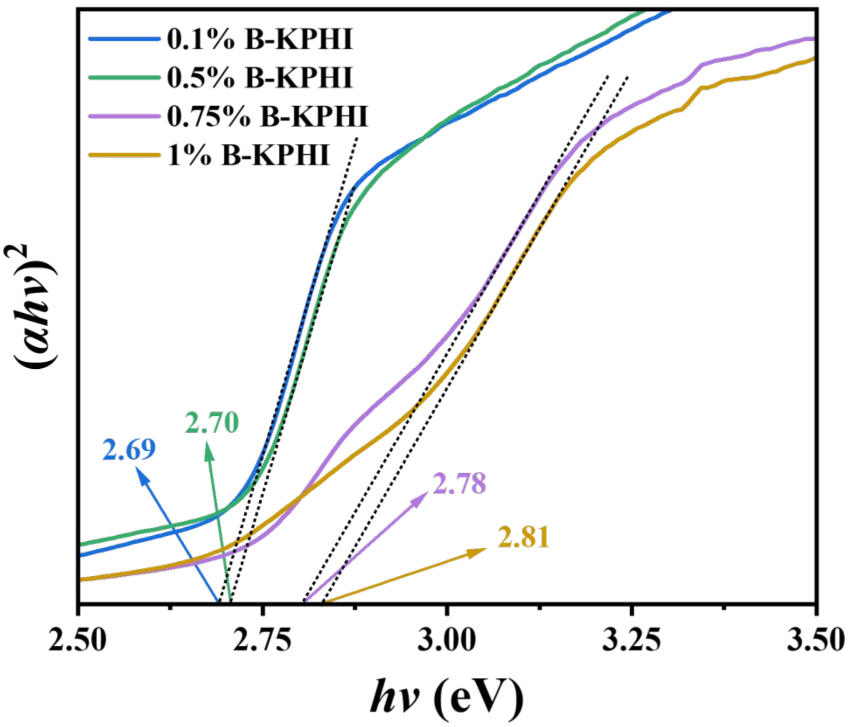

Figure S15. Tauc-plot for determining the optical band gap (E_g) for *x%*B-KPHI catalysts.

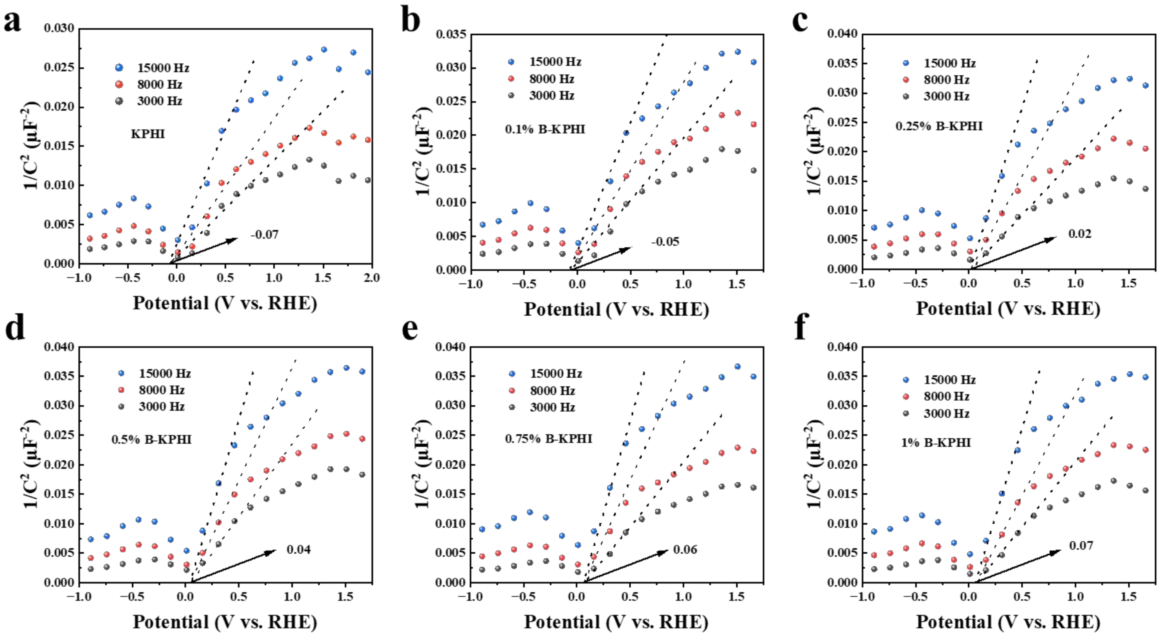

Figure S16. Mott-Schottky plots for KPHI and *x%*B-KPHI catalysts.

Table S7. Distances in Å between K and B-doping atoms for the B-KPHI periodic systems considered in this work.

**K numbers**	**1st K^{+}**		**2nd K^{+}**	
**Boron sites**	**B_1**	**B_2**	**B_1**	**B_2**
**B_{1f}**-KPHI	9.34	-	-	-
**B_{1c}**-KPHI	3.77	-	-	-
**B_{2f}**-KPHI	-	7.13	-	-
**B_{2c}**-KPHI	-	2.99	-	-
**B_{12f}**-KPHI	9.36	7.23	12.23	8.80
**B_{12c}**-KPHI	7.08	2.95	9.59	8.69

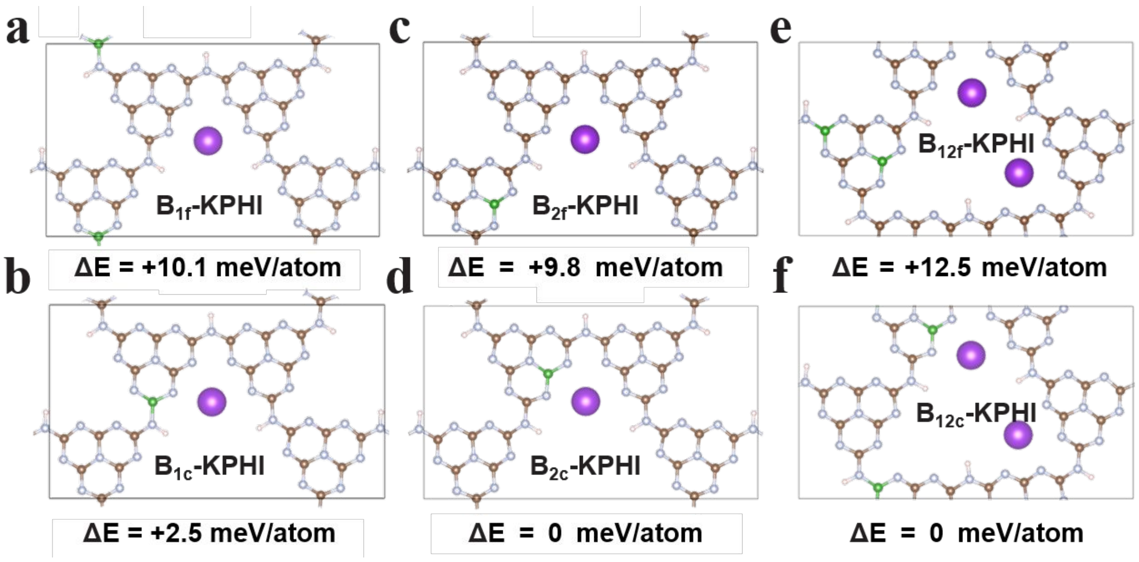

Figure S17. Top view of the KPHI unit cells doped with (a-d) one site and (e-f) two sites of B atoms, where B and K atoms are lying far (top) or close (bottom) to each other, respectively. Their respective relative energy with respect the most stable structure in meV/atom are indicated below each view.

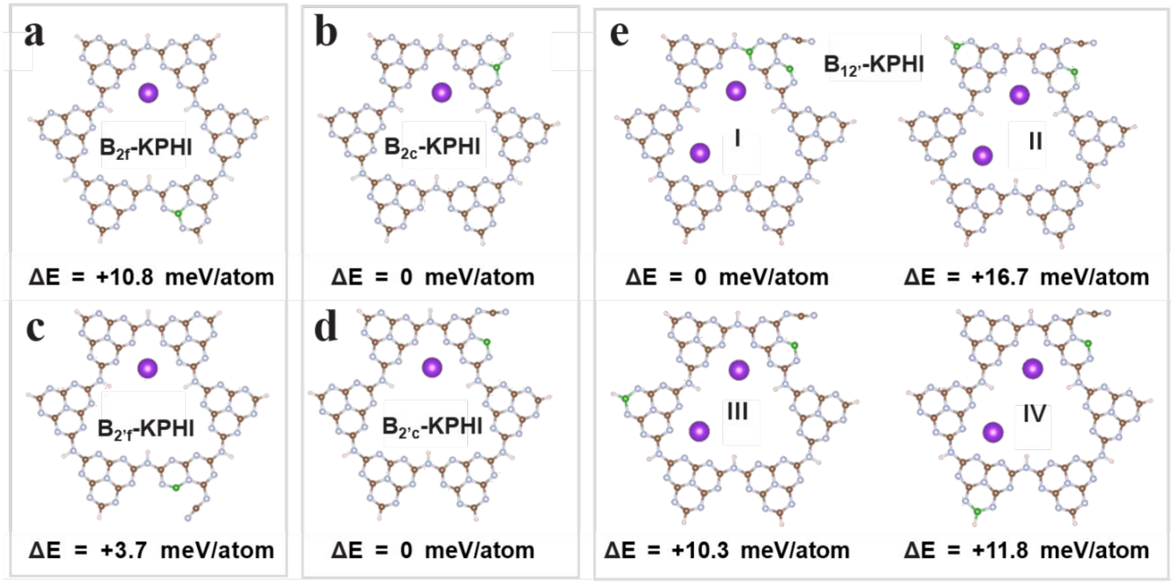

Figure S18. Top views of the defective B-KPHI clusters doped with (a-d) one site and (e) two sites of B-atoms with their corresponding relative energy with respect to their respective most stable cluster structure. Note that the cluster represented in I-panel corresponds to the one whose results are discussed in this work, also denoted as $B_{12'}$-KPHI.

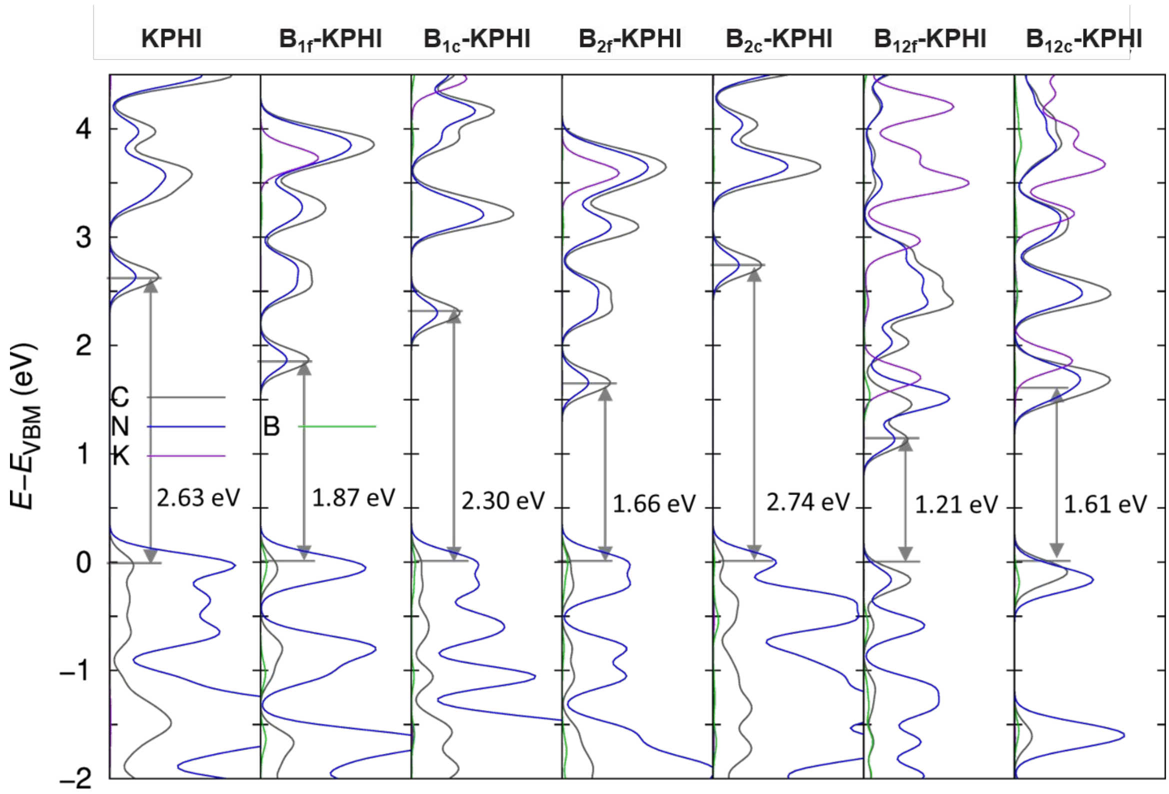

Figure S19. Projected density of states (PDOS) for the atoms constituting the unit cells depicted in Figure S15, where the valence band maximum (VBM) energy is set as energy reference. Vertical arrows are used to represent their electronic gap.

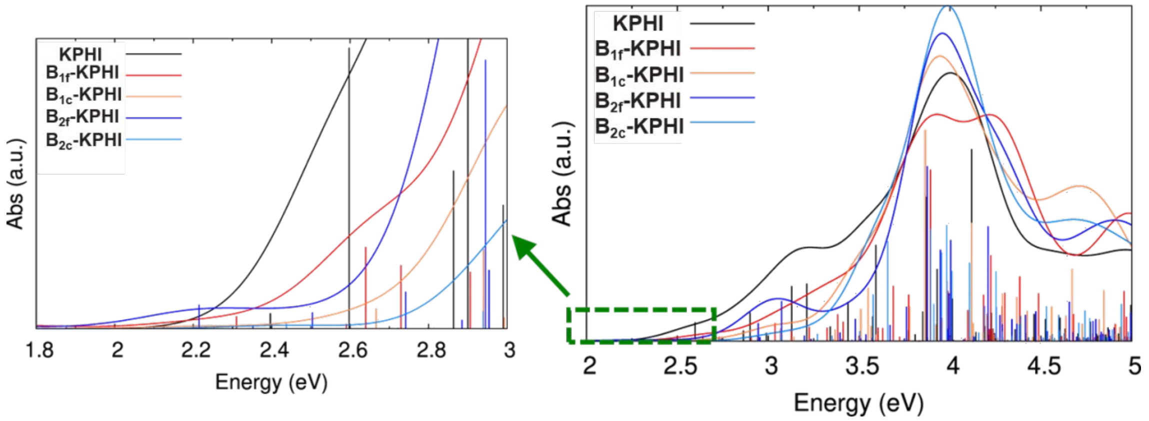

Figure S20. TD-DFPT simulated absorption spectra for the KPHI and *B*-KPHI with one site of B doping periodic structures, as estimated at the TDA-B3LYP/DZVP level, together with the zoomed spectra of the lowest energy region delimited by the green dashed rectangle, on the left of the spectra. Vertical lines correspond to the states composing the spectra and their intensities are related with the oscillator strengths. The characteristics of the most important excited states from these spectra are reported in Table S9.

Table S8. Excited states properties for the main vertical excitations in the lowest energy region of the KPHI and B-KPHI periodic structures: excited state number (*n*), exciton energies (E_x), oscillator strength (*f*), and main occupied-virtual crystalline orbital transitions involved in the excitation, with their corresponding weight coefficients (C_i).

System	*n*	E_x (eV)	*f* (10^{-2} a.u.)	*occupied → virtual*	C_i
**KPHI**	3	2.60	9.68	H-1→L	0.97
	11	3.13	28.30	H-2→L+1	0.66
	14	3.21	29.60	H-1→L+3	0.63
	27	3.59	49.50	H-8→L	0.88
	38	3.87	74.30	H-16→L	0.58
	47	4.12	98.70	H-6→L+2	0.54
**B_{1f}-KPHI**	2	1.70	0.19	H-1→L	0.97
	10	2.64	2.81	H-4→L	0.87
	16	3.04	9.29	H-1→L+4	0.93
	49	3.89	88.20	H-18→L	0.89
**B_{1c}-KPHI**	2	2.25	0.12	H-1→L	0.99
	11	3.07	6.41	H-4→L	0.76
	20	3.37	5.96	H-12→L	0.94
	34	3.86	109.00	H-14→L	0.58
**B_{2f}-KPHI**	5	2.21	0.83	H-1→L+1	0.98
	12	2.74	1.28	H-1→L+3	0.90
	19	3.07	20.50	H-5→L	0.96
	49	3.87	90.00	H-17→L	0.78
**B_{2c}-KPHI**	1	2.44	0.17	H→L	0.99
	8	3.07	3.92	H-7→L	0.61
	24	3.66	51.40	H-8→L	0.91
	34	3.98	59.50	H-4→L+3	0.66

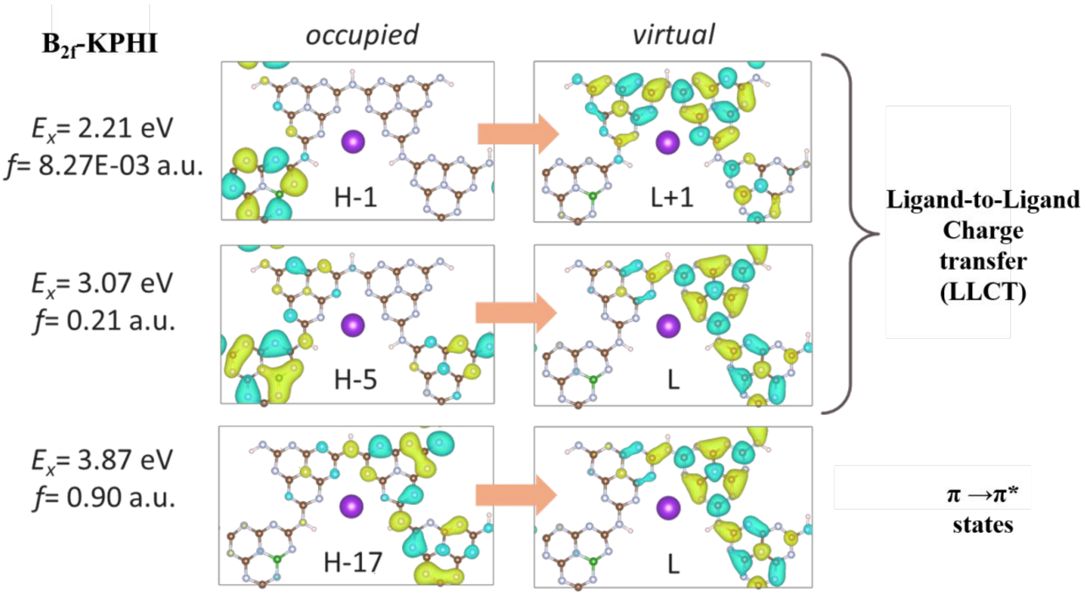

Figure S21. Top view of the unit cells representing the **hole (left)** and **electron (right)** distribution corresponding to the most important occupied and virtual crystalline orbitals involved in the main transitions of B_{2f}-KPHI. The excited state characteristics and occupied and virtual crystalline orbitals for the main vertical transitions at the absorption band edge are reported in Table S9. The iso-value used to plot the iso-density shapes was set to 0.02 a.u.

Table S9. Exciton binding energies (E_b) in meV for the KPHI and B-KPHI bulk materials, as estimated at the at the TDA-B3LYP/DZVP level.

System	E_b (meV)
**KPHI**	306
**B_{1f}-KPHI**	301
**B_{1c}-KPHI**	282
**B_{2f}-KPHI**	308
**B_{2c}-KPHI**	305

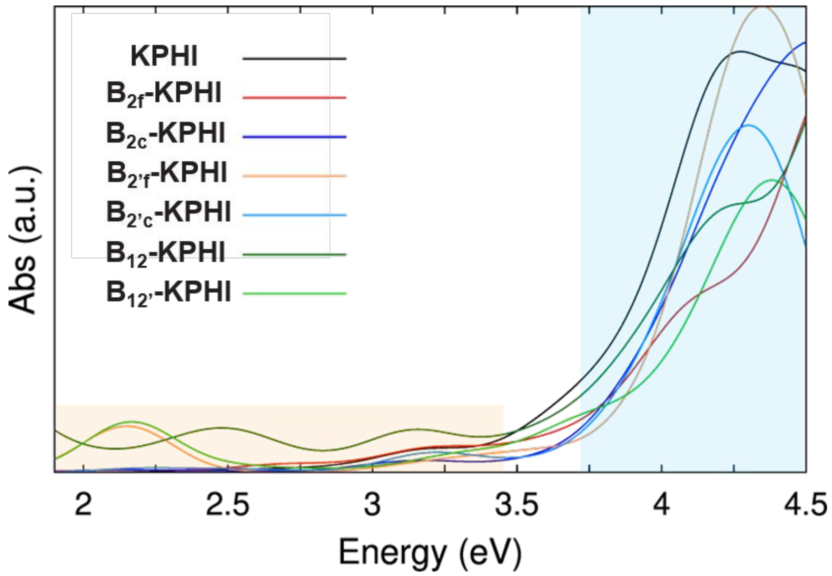

Figure S22. TD-DFT simulated absorption spectra for the KPHI and B-KPHI clusters represented in Figure S16, as calculated at the TDA-B3LYP/DGDZVP level. Orange and blue colors are used to delimit the lowest and higher (> 3.3 eV) absorption energy regions. The characteristics of the vertical excitations conforming these spectra are collected in Table S11.

Table S10. Excited states properties for the main vertical excitations in the lowest energy region of the KPHI and B-KPHI pristine and defective cluster models: excited state number (*n*), exciton energies (E_x), wavelengths (λ) and oscillator strength (*f*).

System	*n*	λ (nm)	E_x (eV)	*f*
**KPHI**	2	503	2.46	0.023
	23	381	3.26	0.064
	49	334	3.71	0.252
	110	292	4.24	0.894
**B_{2f}-KPHI**	28	566	2.19	0.008
	52	461	2.69	0.027
	137	332	3.74	0.083
	203	297	4.18	0.381
**B_{2c}-KPHI**	2	586	2.12	0.023
	24	381	3.25	0.039
	64	318	3.90	0.257
	98	295	4.20	0.496
**$B_{2'f}$-KPHI**	16	572	2.17	0.246
	68	345	3.59	0.069
	119	310	4.00	0.201
	159	295	4.20	0.356
**$B_{2'c}$-KPHI**	13	560	2.21	0.037
	52	382	3.25	0.074
	141	315	3.93	0.138
	192	300	4.14	0.529
**B_{12}-KPHI**	40	511	2.42	0.124
	79	399	3.11	0.076
	165	315	3.93	0.371
	203	295	4.20	0.471
**$B_{12'}$-KPHI**	13	588	2.11	0.173
	14	567	2.19	0.195
	90	338	3.66	0.140
	152	304	4.08	0.450

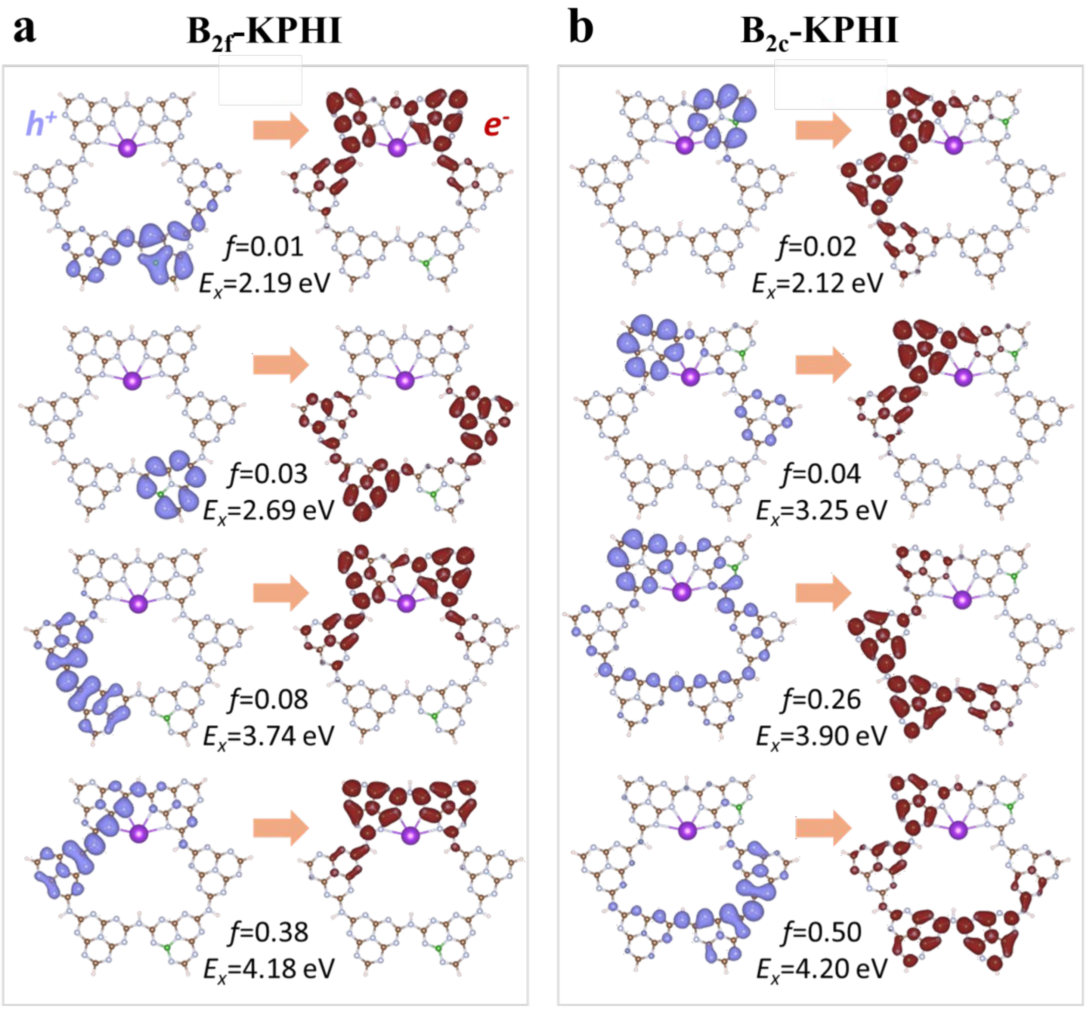

Figure S23. NTO plots for the excited states of the pristine B-KPHI clusters (a) B_{2f}-KPHI and (b) B_{2c}-KPHI reported in Table S11, as calculated at the TDA-B3LYP/DGDZVP level, together with their corresponding absorption energies and oscillator strengths. Purple/red colors are used to depict the occupied/virtual NTO iso-densities. The iso-value density used in the plots was set to 0.02 a.u.

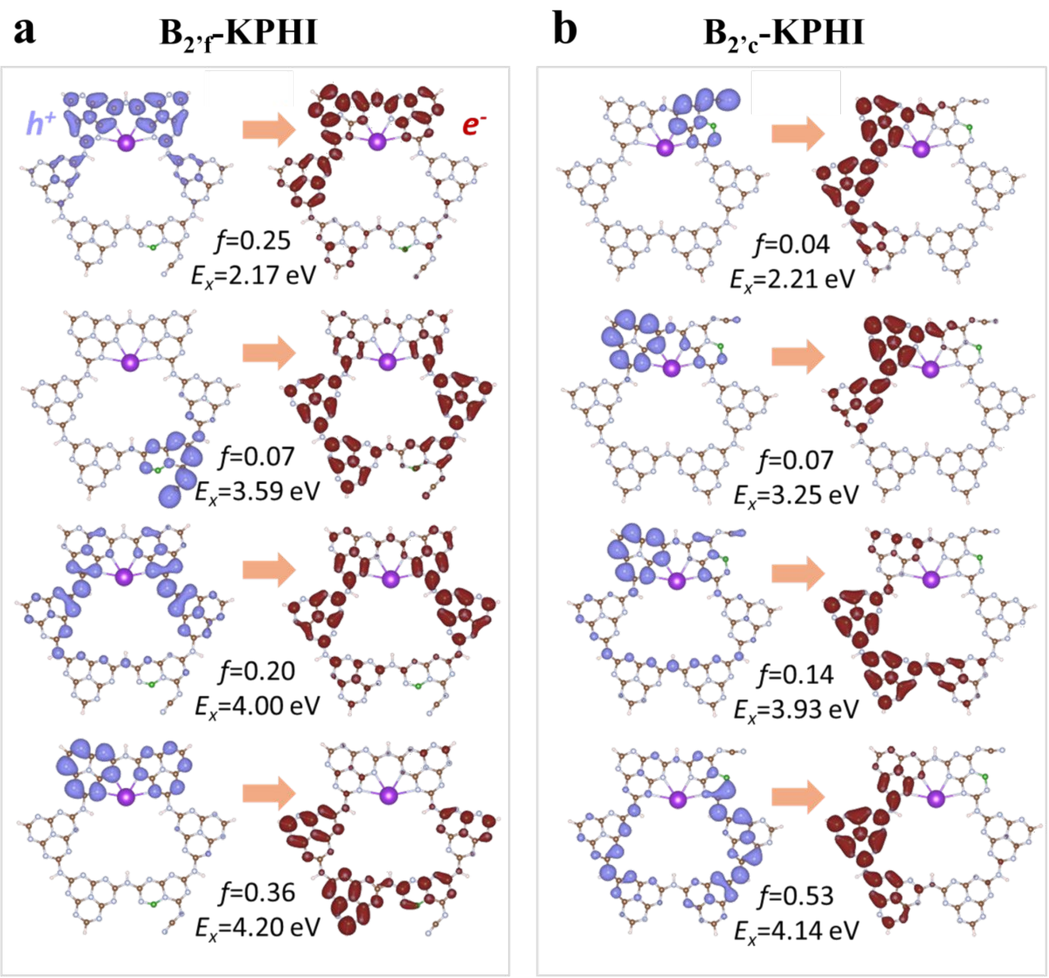

Figure S24. NTO plots for the excited states of the defective B-KPHI clusters (a) $B_{2'f}$-KPHI and (b) $B_{2'c}$-KPHI reported in Table S11, as calculated at the TDA-B3LYP/DGDZVP level, together with their corresponding absorption energies and oscillator strengths. Purple/red colors are used to depict the occupied/virtual NTO iso-densities. The iso-value density used in the plots was set to 0.02 a.u.

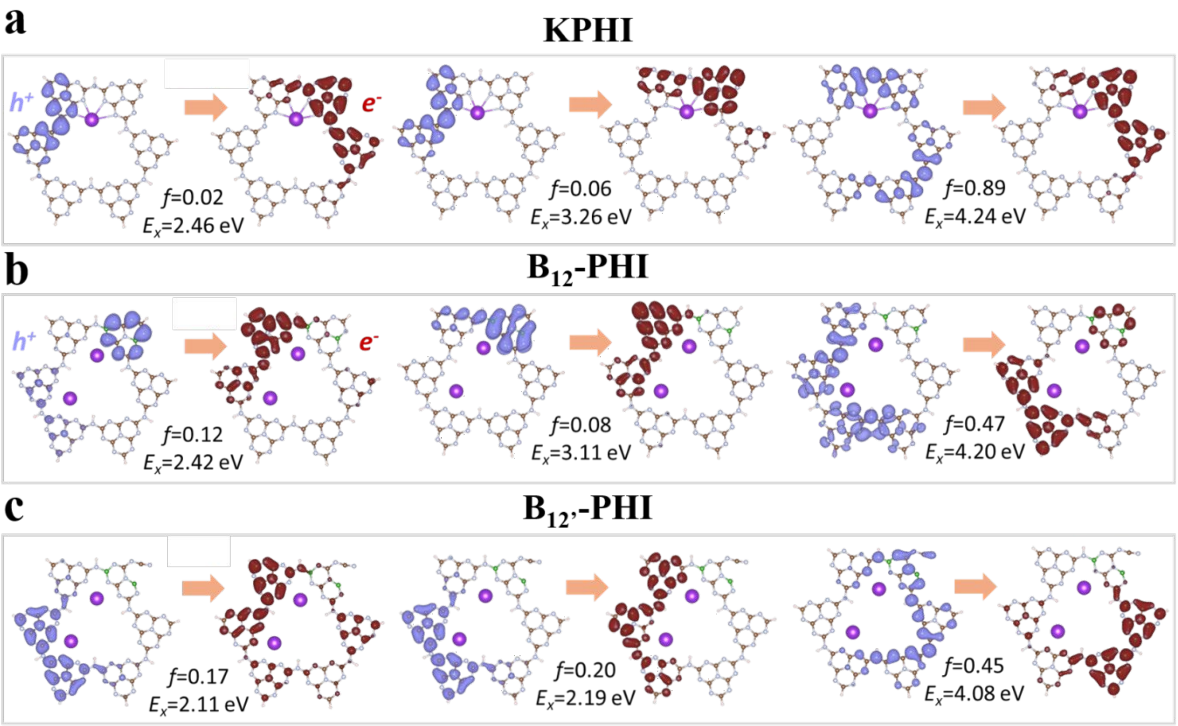

Figure S25. NTO plots for the excited states of pristine (a) KPHI, (b) the two sites of boron doping B_{12}-KPHI and corresponding (c) the defective $B_{12'}$-KPHI clusters reported in Table S11, as calculated at the TDA-B3LYP/DGDZVP level, together with their corresponding absorption energies and oscillator strengths. Purple/red colors are used to depict the occupied/virtual NTO iso-densities. The iso-value density used in the plots was set to 0.02 a.u

Table S11. Adsorption energies (E_{ads}) in eV and equilibrium M*-O_{H2O} distance in Å of the water molecule adsorbed on K* and B* active sites of the defected B-KPHI clusters. E_{ads} have been estimated by the difference between the total energy of the system ($E_{PHI-H2O}$) and the energies of the separated components (E_{PHI} and E_{H2O}) in their ground state geometries.

**System**	***E**_ads_* **(eV)**		***d*(M*-O$_{H2O}$) (Å)**	
	**B***	**K***	**B***	**K***
KPHI	-	-1.29	-	2.653
$B_{2'f}$-KPHI	-2.24	-0.13	1.473	3.193
$B_{2'c}$-KPHI	-2.15	-0.80	1.472	2.764
$B_{12'}$-KPHI	-1.80	-	1.479	-

Table S12. Gibbs free energy changes of each reaction step of the OER calculated for of the defective B-KPHI clusters. The values in red color are used to highlight the overpotential corresponding to the rate determining step of the reaction.

**ΔG (eV)**	**KPHI**	**$B_{2'c}$-KPHI**		**$B_{12'}$-KPHI**
**Active sites**	K	B	K	B
* + H_2O→ *OH + H^+ + e^-	1.72	-3.20	-2.02	-1.30
*OH→ *O + H^+ + e^-	0.97	2.51	3.11	2.61
*O + H_2O→*OOH + H^++ e^-	1.83	2.15	1.95	2.09
*OOH→ *O_2+ H^+ + e^-	0.39	3.46	1.88	1.51

References

[1] H. Tong, J. Odutola, J. Song, L. Peng, N. Tkachenko, M. Antonietti, C. M. Pelicano, *Adv. Mater.* **2024**, 36, 2412753.

[2] S. Liu, V. Diez-Cabanes, D. Fan, L. Peng, Y. Fang, M. Antonietti, G. Maurin, *ACS Catal.* **2024**, 14, 2562–2571.

[3] J. P. Perdew, K. Burke, M. Ernzerhof, *Phys. Rev. Lett.* **1996**, 77, 3865–3868.

[4] S. Grimme, S. Ehrlich, L. Goerigk, *J. Comput. Chem.* **2011**, 32, 1456–1465.

[5] P. E. Blöchl, *Phys. Rev. B* **1994**, 50, 17953–17979.

[6] G. Kresse, D. Joubert, *Phys. Rev. B* **1999**, 59, 1758–1775.

[7] M. Iannuzzi, T. Chassaing, T. Wallman, J. Hutter, *Chimia (Aarau).* **2005**, 59, 499–503.

[8] J. VandeVondele, J. Hutter, *J. Chem. Phys.* **2007**, 127, 114105.

[9] S. Goedecker, M. Teter, J. Hutter, *Phys. Rev. B Condens. Matter.* **1996**, 54, 1703–1710.

[10] J. Hutter, M. Iannuzzi, F. Schiffmann, J. Vandevondele, *WIREs Comput. Mol. Sci.* **2014**, 4, 15–25.

[11] C. Sosa, J. Andzelm, B. C. Elkin, E. Wimmer, K. D. Dobbs, D. A. Dixon, *J. Phys. Chem.* **1992**, 96, 6630–6636.

[12] S.M. Dancoff, *Phys. Rev.* **1950**, 78, 382–385.

[13] I. C. Man, H. Y. Su, F. Calle-Vallejo, H. A. Hansen, J. I. Martínez, N. G. Inoglu, J. Kitchin, T. F. Jaramillo, J. K. Nørskov, J. Rossmeisl, *ChemCatChem* **2011**, 3, 1159–1165.

[14] J. K. Nørskov, J. Rossmeisl, A. Logadottir, L. Lindqvist, J. R. Kitchin, T. Bligaard, H. Jónsson, *J. Phys. Chem. B* **2004**, 108, 17886–17892.

[15] M. J. Frisch, G. W. Trucks, H. B. Schlegel, G. E. Scuseria, M. A. Robb, J. R. Cheeseman, G. Scalmani, V. Barone, G. A. Petersson, H. Nakatsuji, X. Li, M. Caricato, A. Marenich, J. Bloino, B. G. Janesko, R. Gomperts, B. Mennucci, H. P. Hratchian, J. V. Ortiz, A. F. Izmaylov, J. L. Sonnenberg, D. Williams-Young, F. Ding, F. Lipparini, F. Egidi, J. Goings, B. Peng, A. Petrone, T. Henderson, D. Ranasinghe, V. G. Zakrzewski, J. Gao, N. Rega, G. Zheng, W. Liang, M. Hada, M. Ehara, K. Toyota, R. Fukuda, J. Hasegawa, M. Ishida, T. Nakajima, Y. Honda, O. Kitao, H. Nakai, T. Vreven, K. Throssell, J. J. A. Montgomery, J. E. Peralta, F. Ogliaro, M. Bearpark, J. J. Heyd, E. Brothers, K. N. Kudin, V. N. Staroverov, T. Keith, R. Kobayashi, J. Normand, K. Raghavachari, A. Rendell, J. C. Burant, S. S. Iyengar, J. Tomasi, M. Cossi, J. M. Millam, M. Klene, C. Adamo, R. Cammi, J. W. Ochterski, R. L. Martin, K. Morokuma, O. Farkas, J. B. Foresman, D. J. Fox, Gaussian Inc., Wallingford CT (**2016**) Gaussian 09, Revision A.02.

[16] C. Ye, J. X. Li, Z. J. Li, X. B. Li, X. B. Fan, L. P. Zhang, B. Chen, C. H. Tung, L. Z. Wu, *ACS Catal.* **2015**, 5, 6973–6979.

[17] J. S. Zhang, M. Grzelczak, Y. D. Hou, K. Maeda, K. Domen, X. Z. Fu, M. Antonietti, X. C. Wang, *Chem. Sci.* **2012**, 3, 443-446.

[18] L. Z. Zhang, C. Yang, Z. L. Xie, X. C. Wang, *Appl. Catal. B Environ. Energy* **2018**, 224, 886-894.

[19] Z. X. Qin, Y. B. Chen, Z. X. Huang, J. Z. Su, L. J. Guo, *J. Mater. Chem. A* **2017**, 5, 19025-19035.

[20] D. M. Zhao, C. L. Dong, B. Wang, C. Chen, Y. C. Huang, Z. D. Diao, S. Z. Li, L. J. Guo, S. H. Shen, *Adv. Mater.* **2019**, 31, 1903545.

[21] A. Y. Shi, H. H. Li, S. Yin, Z. L. Hou, J. Y. Rong, J. C. Zhang, Y. H. Wang, *Applied Catalysis B: Environmental* **2018**, 235, 197-206.

[22] F. Yu, T. T. Huo, Q. H. Deng, G. A. Wang, Y. G. Xia, H. P. Li, W. G. Houa, *Chem. Sci.* **2022**, 13, 754-762.

[23] F. T. He, S. L. Wang, Y. M. Lu, P. Dong, Y. Zhang, F. F. Lin, X. M. Liu, Y. Q. Wang, C. C. Zhao, S. J. Wang, X. G. Duan, J. Q. Zhang, S. B. Wang, *Nano Energy* **2023**, 116, 108800.
